# Their own worst enemy? Collective narcissists are willing to conspire against their in‐group

**DOI:** 10.1111/bjop.12569

**Published:** 2022-05-06

**Authors:** Mikey Biddlestone, Aleksandra Cichocka, Michał Główczewski, Aleksandra Cislak

**Affiliations:** ^1^ Department of Psychology University of Cambridge Cambridge UK; ^2^ School of Psychology University of Kent Kent UK; ^3^ Institute of Psychology Nicolaus Copernicus University in Toruń Toruń Poland; ^4^ Institute of Psychology SWPS University of Social Sciences and Humanities Warszawa Poland

**Keywords:** collective narcissism, conspiracy beliefs, conspiracy theories, in‐group identification, populism

## Abstract

Collective narcissism – a belief in in‐group greatness that is not appreciated by others – is associated with using one's group for personal benefits. Across one pilot and four studies, we demonstrated that collective narcissism predicts readiness to conspire against in‐group members (*r*_*meta‐analysis*_ = .24). In Study 1, conducted in Poland (*N* = 361), collective narcissism measured in the context of national identity predicted readiness to engage in secret surveillance against one's own country's citizens. In Study 2 (*N* = 174; pre‐registered), collective narcissism in UK workplace teams predicted intentions to engage in conspiracies against co‐workers. In Study 3 (*N* = 471; pre‐registered), US national narcissism predicted intentions to conspire against fellow citizens. Furthermore, conspiracy intentions accounted for the relationship between collective narcissism and beliefs in conspiracy theories about the in‐group. Finally, in Study 4 (*N* = 1064; pre‐registered), we corroborated the link between Polish national narcissism and conspiracy intentions against fellow citizens, further showing that these intentions were only directed towards group members that were perceived as moderately or strongly typical of the national in‐group (but not when perceived in‐group typicality was low). In‐group identification was either negatively related (Studies 1 and 2) or unrelated (Studies 3 and 4) to conspiracy intentions (*r*_*meta‐analysis*_ = .04). We discuss implications for research on conspiracy theories and populism.

**Practitioner points**

- Analysts should monitor cases of public endorsement of collective narcissism, which is a belief that one’s in‐group (e.g. nation, organisation, or political party) is exceptional but underappreciated by others.
- As we show, collective narcissism is associated with a willingness to conspire against fellow in‐group members and with support for in‐group surveillance policies.
- Thus, groups cherishing such a defensive form of in‐group identity are threatened from the inside, thereby warranting education aimed at identifying and avoiding potential exploitation from otherwise trusted members within their own groups.

## BACKGROUND

From 1953 until 1973, the CIA carried out secret illegal human experiments on US citizens and military personnel to gain a better understanding of how to control individuals through psychological torment and torture (see Kinzer, 2019). These often took place in overseas detention camps to avoid criminal prosecution. While moral justifications for developing methods of espionage are far from straightforward, the vast human suffering and death caused by these practices raise important questions about the extent to which group members might be willing to collude against their own in‐group to achieve distal goals. While there is extensive research on the psychological concomitants of *conspiracy beliefs* (Douglas et al., 2017; Douglas et al., 2019), less is known about the processes that might explain personal willingness to become involved in conspiracies (cf., Douglas & Sutton, 2011). We aim to address this gap in the current research, focusing especially on the role of group identity.

### Who might be ready to conspire?

Literature on willingness to conspire is scarce. One exception is research by Douglas and Sutton (2011), who showed that individuals were more likely to believe conspiracy theories if they were personally willing to engage in conspiracies. For example, those who believed in 9/11 conspiracy theories were more likely to say they would have ordered the attack on the Twin Towers themselves. Moreover, an experimental manipulation of personal morality reduced participants' conspiracy theory endorsement, but only indirectly through a reduction of their personal willingness to conspire. As a result, the authors concluded that this may be an example of projection (Ames, 2004; McClosky, 1958) – a process whereby individuals attribute their own thoughts, feelings and motivations onto others in order to make sense of their social environment.

In their work, Douglas and Sutton (2011) also examined personality predispositions related to conspiring. They found that intentions to conspire were associated with Machiavellianism – a personality trait measuring willingness to exploit others for selfish gains (Festinger & Schachter, 1970; Paulhus & Williams, 2002; see also Uscinski et al., 2021). However, conspiring is rarely only an interpersonal endeavour. Most conspiracy theories presume that a powerful group is colluding to further their own agenda (Lewandowsky et al., 2013) and harm one's in‐group (Biddlestone et al., 2020; Cichocka, Marchlewska, Golec de Zavala et al., 2016; Sternisko et al., 2020; van Prooijen & van Lange, 2014). We seek to examine whether the way people feel about their groups might also be associated with conspiratorial intentions. We argue that those high in collective narcissism – that is, those who tend to use their group for personal gains (Cichocka et al., 2021; Cichocka & Cislak, 2020; Marchlewska et al., 2020) – might be willing to engage in conspiracies against other group members to achieve their own selfish goals. Below, we discuss past work on collective narcissism, highlighting its implications for conspiracy beliefs, followed by our theoretical rationale for why this form of in‐group identity might also predict intentions to engage in conspiracies.

### The psychological and political concomitants of collective narcissism

People can identify with their social groups in different ways. They might feel happy about their group membership and connected to other group members. We can call this a ‘conventional’ in‐group identification (Cameron, 2004; Leach et al., 2008; Tajfel & Turner, 1979). However, people can also be defensive about their social identities (Cichocka, 2016) – they might believe that their in‐group is exceptional but is not getting the recognition it is entitled to. Such beliefs are captured by the concept of collective narcissism (Golec de Zavala et al., 2009). Collective narcissism is related to other measures of excessive in‐group identity, such as nationalism (Golec de Zavala et al., 2016; Kosterman & Feshbach, 1989; Lyons et al., 2010) or in‐group glorification (Roccas et al., 2006). However, collective narcissism can be seen as a broader underlying defensive need for in‐group recognition, which can be measured beyond the national context (but see also Roccas et al., 2008). For example, it can refer to religion (Marchlewska et al., 2019; Yustisia et al., 2019), sports teams (Larkin & Fink, 2019) or extremist organizations (Jasko et al., 2020).

Collective narcissism is thought to be generally motivated by a frustration of basic needs (Cichocka, 2016; Fromm, 1973), such as the need for personal control (Cichocka et al., 2018; Marchlewska et al., 2020) or self‐worth (Golec de Zavala et al., 2019). In line with its compensatory nature, collective narcissism is linked to defensive tendencies to perceive outgroups as threatening (Golec de Zavala et al., 2016) and to respond to threats with outgroup derogation or aggression (e.g. Golec de Zavala, Cichocka, & Iskra Golec, 2013; Lyons et al., 2010). This threat sensitivity explains why collective narcissism predicts susceptibility to conspiracy theorizing about outgroups (e.g. Cichocka, Marchlewska, Golec de Zavala et al., 2016; Golec de Zavala & Cichocka, 2012).

#### Collective narcissism and intergroup conspiracy beliefs

Multiple studies show links between collective narcissism (but not in‐group identification) and conspiracy beliefs. Across various countries, national narcissism (i.e. collective narcissism in reference to one's nation) predicted the endorsement and dissemination of COVID‐19 conspiracy theories (Sternisko et al., 2021). In Poland, national narcissism predicted beliefs in conspiracies about foreign involvement in high‐profile events, such as the Smolensk crash (Cichocka, Marchlewska, Golec de Zavala et al., 2016; Soral et al., 2018). In a religious context, Marchlewska and colleagues (2019) found that catholic narcissism predicted belief in a so‐called gender conspiracy, which assumes that ‘gender studies and gender‐equality activists…secretly promote an ideology designed to harm traditional values and social arrangements’ (p. 766).

These findings suggest that collective narcissism is linked to conspiracy beliefs about outgroups. However, it has rarely been associated with belief in in‐group conspiracies. Cichocka, Marchlewska, Golec de Zavala and colleagues (2016) found that while US national narcissism predicted convictions that foreign governments are conspiring, it was not associated with similar convictions about own governments. However, Golec de Zavala and Federico (2018) showed that national narcissism can be associated with a more general conspiratorial mindset – predicting a rise in generalized conspiratorial thinking over the course of the 2016 US election. This finding suggests that collective narcissism might not only be predictive of belief in outgroup conspiracies, but also of a more general tendency to believe in a Manichean distinction between ‘us’ and a malevolent ‘them’, even within one nation (see also Uscinski et al., 2016, 2021). Thus, it seems plausible that collective narcissism may be associated with belief in conspiracies within the group, perhaps offering another target to blame for the insufficient recognition of the in‐group's greatness (see also Marques & Paez, 1994).

Work demonstrating the robust associations between collective narcissism and conspiracy beliefs can help explain why certain political movements are especially drawn to conspiracy theories. Studies show that national narcissism is a predictor of support for national populist parties and politicians (Cislak et al., 2020; Federico & Golec de Zavala, 2018; Marchlewska et al., 2018; Forgas & Lantos, 2019), who allegedly defend the ‘real people’ from the malevolent national elites and often accuse others of conspiring (Bergmann, 2018; Müller, 2016; van Prooijen, 2018; see also Castanho Silva et al., 2017). Ironically, leaders of populist movements themselves have also been caught engaging in conspiratorial behaviours. For example, the Polish government withheld information when questioned about their secret purchasing of a surveillance technology called *Pegasus*, which would allow them to spy on citizens without their knowledge (Reuters, 2022). President Trump was under investigation for potential collusion with Russia to interfere in the 2016 election (Weiss, 2017). We argue that collective narcissism might be a factor that explains not only a tendency to perceive conspiracies within and beyond the group, but also a willingness to conspire against fellow group members.

#### Collective narcissism and a willingness to conspire against the in‐group

Recent theorizing on collective narcissism suggests that it is associated with selfish motivations (Cichocka & Cislak, 2020; Marchlewska et al., 2020). Because collective narcissism compensates for frustrated personal needs (Bertin et al., 2022; Cichocka et al., 2018), it is linked to a greater preoccupation with how the group image reflects on the individual and using the group to achieve selfish goals, than with benefiting other in‐group members (Cichocka, 2016). This lack of genuine commitment to in‐group members might carry potentially problematic consequences for the in‐group. For example, national narcissism predicted a greater willingness to leave one's own country for financial gains (Marchlewska et al., 2020) and supporting policies that could harm the in‐group in the long run (e.g. problematic public health policies; Cislak, Marchlewska, et al., 2021; Gronfeldt et al., 2022; or anti‐conservation policies; Cislak et al., 2018; Cislak, Cichocka et al., 2021). In the workplace context, organizational narcissism predicted treating in‐group members instrumentally (i.e. using them for personal benefits; Cichocka et al., 2021).

These findings hint at why those scoring high in collective narcissism may be willing to conspire against their in‐group members: they might be especially likely to use their group to achieve their objectives. Indeed, conspiracy theories assume that people are colluding to further their own selfish agenda (Lewandowsky et al., 2013). Furthermore, in‐group members may be relatively easier to take advantage of than outgroup members because they tend to be more trusting and expect fairness (Foddy et al., 2009), but also because there is simply more information available about them to draw upon (see also Cichocka et al., 2021). Thus, plotting against in‐group members may seem even less costly and more profitable for the self than plotting against outgroup members. Furthermore, those scoring high in collective narcissism may project their selfish motivations onto other in‐group members. This, in turn, might increase the perceived threat that even in‐group members pose, thus making them a potential target of conspiracy theories. Following theorizing by Douglas and Sutton (2011), we argue that a personal willingness to conspire against in‐group members may be accompanied by belief in in‐group conspiracies.

Collective narcissism assumes a positive in‐group evaluation and, hence, correlates with conventional in‐group identification (Golec de Zavala et al., 2009). However, the two tend to be associated with different outcomes, especially once their shared variance is accounted for. For example, while collective narcissism predicts greater outgroup prejudice, in‐group identification (without the narcissistic component) predicts greater outgroup tolerance (Golec de Zavala, Cichocka, & Bilewicz, 2013). In‐group identification has also been associated with more positive outcomes for the in‐group, including greater loyalty (Marchlewska et al., 2020), and lower likelihood of exploiting in‐group members (Cichocka et al., 2021). Thus, we would expect in‐group identification to predict lower readiness to conspire against in‐group members.

### Overview of the current studies

In the present studies, we investigate predictors of a readiness to conspire against in‐group members. We argue that because collective narcissism is associated with using in‐group members for personal benefits, it might also be positively associated with intentions to conspire against them. We also test whether intentions to conspire against the in‐group might indirectly link collective narcissism to belief in in‐group conspiracies. All studies used a cross‐sectional design, measuring collective narcissism, in‐group identification and conspiracy intentions against the in‐group in various contexts.

In Studies 1 and 4, we investigated whether Polish national narcissism was associated with personal intentions to conspire against other Polish citizens by secretly spying on them. In Studies 2 and 3, we examined intentions to engage in a wider range of conspiracies. In Study 2, conducted in the United Kingdom, we examined the associations between organizational narcissism and conspiracy intentions in a workplace context. In Study 3, conducted in the United States, we again focused on the national context and measured national narcissism, identification and intentions to engage in even more varied conspiracies against fellow US citizens. We also included a measure of in‐group conspiracy beliefs to determine whether conspiracy intentions mediate the relationship between national narcissism and belief in in‐group conspiracy theories. In Study 4, we investigated the link between Polish national narcissism and conspiracy intentions, adding a measure of perceived in‐group typicality to explore exactly which in‐group members those high in national narcissism might be willing to conspire against. Because conspiracy intentions are likely to be positively skewed, in all studies we conducted our analyses with the use of bias‐corrected bootstrapping (with 1000 resamples).^1^ Data and pre‐registration documents are posted at https://osf.io/gxytw/.

## STUDY 1

In Study 1, we examined whether national narcissism and identification would predict individuals' intentions to engage in conspiracies against citizens of their own nation. We focused on conspiracies involving surveillance. Ideas of secret citizen monitoring feature frequently in modern conspiracy theories (e.g. Bruder et al., 2013). For example, one conspiracy theory argues that COVID‐19 vaccines contain surveillance microchips (Romano, 2020). Although surveillance may not be considered a conspiracy in itself, *secret* surveillance without the consent of the individuals being monitored often carries an implicit conspiratorial intention. In fact, reflecting in *The Guardian* on his role as a whistle‐blower against the US National Security Agency, Edward Snowden (2021) referred to mass surveillance as a ‘true conspiracy’ (para. 4). A pilot study, conducted in Poland, established that national narcissism (but not national identification) predicted support for the Polish government's covert use of a surveillance software against Polish citizens (see Appendix S1). In Study 1, we examined whether national narcissism would also predict a willingness to personally engage in secret surveillance.

### Method

#### Participants and design

Study 1 was part of a larger survey,^2^ completed by 361 Polish participants, 228 men, 133 women, aged 18–77 (*M*_age_ = 43.11, *SD*_age_ = 14.36). Sensitivity analysis with G*Power (*α* = .05, *β* = .80, two‐tailed) suggested that we had enough power to detect a small effect size of *r* = .15. Participants filled out measures of national narcissism and identification (counterbalanced), and then of conspiracy intentions, political ideology and demographics (age, gender and education). Unless noted otherwise, we used response scales from *definitely not* (1) to *definitely yes* (7).

#### Measures

*National Narcissism* was measured with five items of the Collective Narcissism Scale (e.g. ‘If Poles had a major say in the world, the world would be a much better place’; Golec de Zavala et al., 2009).

*National Identification* was measured with five items (e.g. ‘I feel strong ties to other Polish people’) based on Cameron's (2004) scale.

*Conspiracy Intentions*. Three items were created to measure conspiracy intentions (see Douglas & Sutton, 2011). Participants were asked whether, if they held a position in the government, they would ‘support rapid responses of intelligence agencies’ in the form of ‘wiretapping citizens’, ‘spreading false information if the situation required it’, and ‘performing Internet surveillance without the consent of the observed citizens’.

*Political Ideology* was measured with a single item asking participants to indicate their political orientation on a scale from *definitely left‐wing* (1) to *definitely right‐wing* (7).

### Results and discussion

As hypothesized, the correlation between national narcissism and conspiracy intentions was positive and significant (Table 1). We also observed a significant positive correlation between in‐group identification and conspiracy intentions.

**TABLE 1:** Means, standard deviations, reliabilities and zero‐order correlations (Study 1)

	*M*	*SD*	*α*	1	2	3	4
1. Conspiracy intentions	2.46	1.65	.90	–	.29***	.06	.25***
2. National narcissism	4.45	1.55	.92		–	.55***	.32***
3. National identification	5.23	1.30	.85			–	.20***
4. Political ideology	4.00	1.41	–				–

***p < .001.

When the overlap between national narcissism and identification was accounted for, national identification became a significant *negative* predictor of conspiracy intentions, while the effect of national narcissism remained significant and positive (Table 2).

**TABLE 2:** Regression model with conspiracy intentions as the dependent variable (Study 1)

Variables	*B* [95% CI]	*β*	*p*
National narcissism	0.34 [0.20, 0.47]	.32	<.001
National identification	−0.20 [−0.35, −0.06]	−.16	.007
Political ideology	0.21 [0.07, 0.34]	.18	.002
Political ideology^2^	0.02 [−0.04, 0.08]	.03	.549
*F*	*F*(4, 356) = 13.15, *p* < .001		
*R* ^*2*^_*adj*_	.12		

Because national narcissism tends to be associated with political conservatism (Cichocka et al., 2017; Golec de Zavala et al., 2009), and because we wanted to verify whether conspiracy intentions might be associated with left–right (or extremist; see van Prooijen et al., 2015) political orientation, we also tested linear and quadratic effects of political ideology. We found a significant linear effect of ideology only, suggesting that it was right‐wingers, rather than extremists, that were willing to engage in conspiracies. The associations for national narcissism and national identification remained significant when adjusting for political ideology or demographics. Overall, we demonstrated that Polish national narcissism predicted intentions to conspire against fellow Poles.

## STUDY 2

Despite the clear conspiratorial nature of covert surveillance, it is possible that participants may interpret the intention items we used in Study 1 as solely referring to a benevolent protection of their in‐group. However, benevolent intentions do not necessarily contradict conspiratorial notions. In fact, conspiratorial intentions might be perceived as a benevolent antidote to an unjust world from the conspirator's perspective (see also Moulding et al., 2016). Nevertheless, in Study 2, we sought to test whether our predictions replicate beyond the specific context of national surveillance. Study 2 focused on conspiracies in the workplace. Past research showed that belief in workplace conspiracy theories can have potentially problematic consequences, including lack of commitment or turnover intentions (Douglas & Leite, 2017; van Prooijen & de Vries, 2015). We examined predictors of people's readiness to engage in a range of workplace conspiracies, such as secret surveillance or corrupt employee favouritism.

Study 2 was conducted with UK participants working in teams. We tested the hypothesis that workplace conspiracy intentions would be positively predicted by high team narcissism (but not team identification). The hypotheses, design and analyses were pre‐registered.

### Method

#### Participants and design

A priori power analysis using the average social psychology effect size (*r* = .21; Richard et al., 2003) determined that a sample size of 173 would achieve a power of .80. The survey was completed by 174 participants, 50 men, 124 women, aged 20–68 (*M*_age_ = 37.93, *SD*_age_ = 10.91). Participants filled out measures of collective narcissism and identification in relation to their workplace teams, workplace conspiracy intentions and demographics (age, gender and education).

#### Measures

*Team Narcissism* was measured with five items of the Collective Narcissism Scale (Golec de Zavala, Cichocka, & Bilewicz, 2013), in relation to the team that participants worked in (e.g. ‘Not many people seem to understand the importance of my team’), with responses from *strongly disagree* (1) to *strongly agree* (7).

*Team Identification* was measured with Cameron's (2004) 12‐item identification scale used in reference to one's team (e.g. ‘Being a member of my team is an important reflection of who I am’), with responses from *strongly disagree* (1) to *strongly agree* (7).

*Conspiracy Intentions*. Five items were created to measure workplace conspiracy intentions. Participants read the following introduction ‘Imagine you've learned that some of your friends secretly coordinated to engage in activities that would help you gain advantage over other members of your team. To what extent would you be willing to join the following activities in your workplace…’. They were then presented with five scenarios and asked whether they would ‘promote underperforming but loyal teammates’, ‘spread false information about them’, ‘secretly control other team members' computers’, ‘monitor their web activity’, and ‘record their phone conversations’, with responses from *definitely not* (1) to *definitely yes* (5).

### Results and discussion

Conspiracy intentions were negatively correlated with team identification, but unrelated to team narcissism (Table 3). However, when their shared variance was accounted for in a regression model (Table 4), we found that conspiracy intentions were positively predicted by team narcissism and negatively predicted by team identification. When we additionally controlled for demographics, the positive link for team narcissism became only marginally significant, *B* = 0.10, 95% CI [0.01, 0.19], *β* = .15, *p* = .055 (although note that the standardized effect size remained identical). Thus, findings in the workplace context were less consistent than in the national context.

**TABLE 3:** Means, standard deviations, reliabilities and zero‐order correlations (Study 2)

Variable	*M*	*SD*	*α*	1	2	3
1. Workplace conspiracy intentions	1.56	0.65	.80	–	.10	−.17*
2. Team narcissism	4.18	0.96	.70		–	.23**
3. Team identification	4.58	0.98	.89			–

*p < .05. **p < .01.

**TABLE 4:** Regression model with workplace conspiracy intentions as the dependent variable (Study 2)

Variables	*B* [95% CI]	*β*	*p*
Team narcissism	0.10 [0.01, 0.20]	.15	.044
Team identification	−0.14 [−0.24, −0.05]	−.21	.011
*F*	*F*(2, 171) = 4.58, *p* = .012		
*R* ^*2*^_*adj*_	.04		

## STUDY 3

In Study 3, we again examined political conspiracies, this time in the United States. Despite the more varied conspiracy items in Study 2, most items so far have focused on intentions to conspire through secret surveillance. Therefore, in Study 3, we planned to measure a wider range of conspiratorial scenarios. We also examined whether, in line with Douglas & Sutton (2011), personal willingness to conspire would be associated with belief in in‐group conspiracies. Past work has found mixed evidence for the link between national narcissism and belief in own government conspiracies (Cichocka, Marchlewska, Golec de Zavala et al., 2016; Golec de Zavala & Federico, 2018). We argue that national narcissism may be associated with in‐group conspiracy beliefs positively insofar as it is linked to conspiracy intentions. Thus, we tested the hypotheses that US national narcissism would positively predict intentions to conspire against the national in‐group and that these intentions would positively predict conspiracy beliefs about the national in‐group. The design, hypotheses and analyses were pre‐registered. Given past research linking national narcissism to support for Trump's presidency (Golec de Zavala & Federico, 2018; Marchlewska et al., 2018), we also conducted exploratory analyses investigating the role of support for Trump (vs. Clinton) in these processes.

### Method

#### Participants and design

According to Fritz and MacKinnon (2007), a mediation analysis expecting small effect sizes (*α* = .14; *β* = .14) requires a sample size of 462 to achieve a power of .80. The survey was completed by 471 participants recruited from *Prolific*, 234 men, 229 women (eight other), aged 18–77 (*M*_age_ = 36.94, *SD*_age_ = 12.08). To achieve balance in terms of political ideology, participants were pre‐screened so that about half voted for Trump (*N* = 235), while others voted for Clinton (*N* = 236) in the 2016 election.

Participants completed the national narcissism and identification scales (counterbalanced) and were next randomly presented with belief items and conspiracy intention items (counterbalanced). These items were grouped so that participants did not see both versions of the same item (see Appendix S1). Finally, participants reported political ideology and demographics (age and gender).^3^

#### Measures

*National Narcissism* was measured as in Study 2, referencing the United States as the in‐group, on a scale from *strongly disagree* (1) to *strongly agree* (5).

*National Identification* was measured with Leach and colleagues' (2008) 4‐item in‐group satisfaction sub‐scale (e.g. ‘I think that Americans have a lot to be proud of’), on a scale from *strongly disagree* (1) to *strongly agree* (5).

*Conspiracy Beliefs about the In‐group*. Eight items from the Generic Conspiracist Belief Scale (GCB; Brotherton et al., 2013) were selected because they refer to the government or institutions (e.g. ‘the government is involved in the murder of innocent citizens and/or well‐known public figures, and keeps this a secret’). Participants responded to four items on a scale from *definitely not true* (1) to *definitely true* (5), while the remaining four were used to measure conspiracy intentions.

*Conspiracy Intentions*. We altered the eight selected GCB items to measure intentions to conspire with the US government. For example, the GCB item ‘The government permits or perpetrates acts of terrorism on its own soil, disguising its involvement’ was altered to ‘If it was necessary, I would work with the government to carry out acts of terrorism on my own soil, disguising our involvement’, and ‘The spread of certain viruses and/or diseases is the result of the deliberate, concealed efforts of some organization’ was altered to ‘If asked, I would aid organizations in concealing efforts that could lead to the spread of certain viruses and/or diseases.’ Participants responded to four items on a scale from *I would never do this* (1) to *I would definitely do this* (5), while the remaining four were used to measure beliefs.

*Political Ideology* was measured with a single item: ‘Overall, where would you place yourself, on the following scale of liberalism‐conservatism?’, on a scale from *extremely liberal* (1) to *extremely conservative* (5).

### Results

As hypothesized, the correlation between national narcissism and conspiracy intentions was positive and significant, as was the relationship between conspiracy intentions and in‐group conspiracy beliefs (Table 5). National narcissism did not significantly correlate with in‐group conspiracy beliefs.

**TABLE 5:** Means, standard deviations, reliabilities and zero‐order correlations (Study 3)

	*M*	*SD*	*α*	1	2	3	4	5
1. Conspiracy beliefs	2.99	1.07	.83/.85	–	.13**	−.04	−.18***	−.10*
2. Conspiracy intentions	1.54	0.69	.71		–	.27***	.17***	.13**
3. National narcissism	2.51	0.98	.90			–	.73***	.64***
4. National identification	3.85	1.09	.94				–	.60***
5. Political ideology	2.83	1.26	–					–

*Note*: Reliability of conspiracy beliefs is reported for the two versions of the scale. Reliability of the conspiracy intentions measure was the same in both versions.

*p < .05. **p < .01. ***p < .001.

We then conducted hierarchical regression analyses. In the first model, conspiracy intentions were positively predicted by national narcissism, but not by national identification or political ideology (linear or extremist; Table 6). We tested a similar model with in‐group conspiracy beliefs as the dependent variable (Table 6), showing that these beliefs were positively predicted by national narcissism and negatively predicted by national identification. Political ideology (linear or extremist) did not predict national conspiracy beliefs. This pattern of results remained similar when we controlled for demographics.

**TABLE 6:** Regression model with conspiracy intentions and beliefs as dependent variables (Study 3)

	Conspiracy intentions			Conspiracy beliefs		
Variables	*B* [95% CI]	*β*	*p*	*B* [95% CI]	*β*	*p*
National narcissism	0.24 [0.12, 0.37]	.34	<.001	0.25 [0.08, 0.42]	.23	.004
National identification	−0.03 [−0.11, 0.05]	−.05	.428	−0.32 [−0.48, −0.17]	−.31	<.001
Political ideology	−0.03 [−0.13, 0.06]	−.06	.538	−0.05 [−0.17, 0.07]	−.06	.334
Political ideology^2^	−0.02 [−0.07, 0.02]	−.05	.355	−0.01 [−0.08, 0.07]	−.001	.984
*F*	*F*(4, 466) = 9.87, *p* < .001			*F*(4, 466) = 6.53, *p* < .001		
*R* ^*2*^_*adj*_	.07			.05		

**FIGURE 1:**
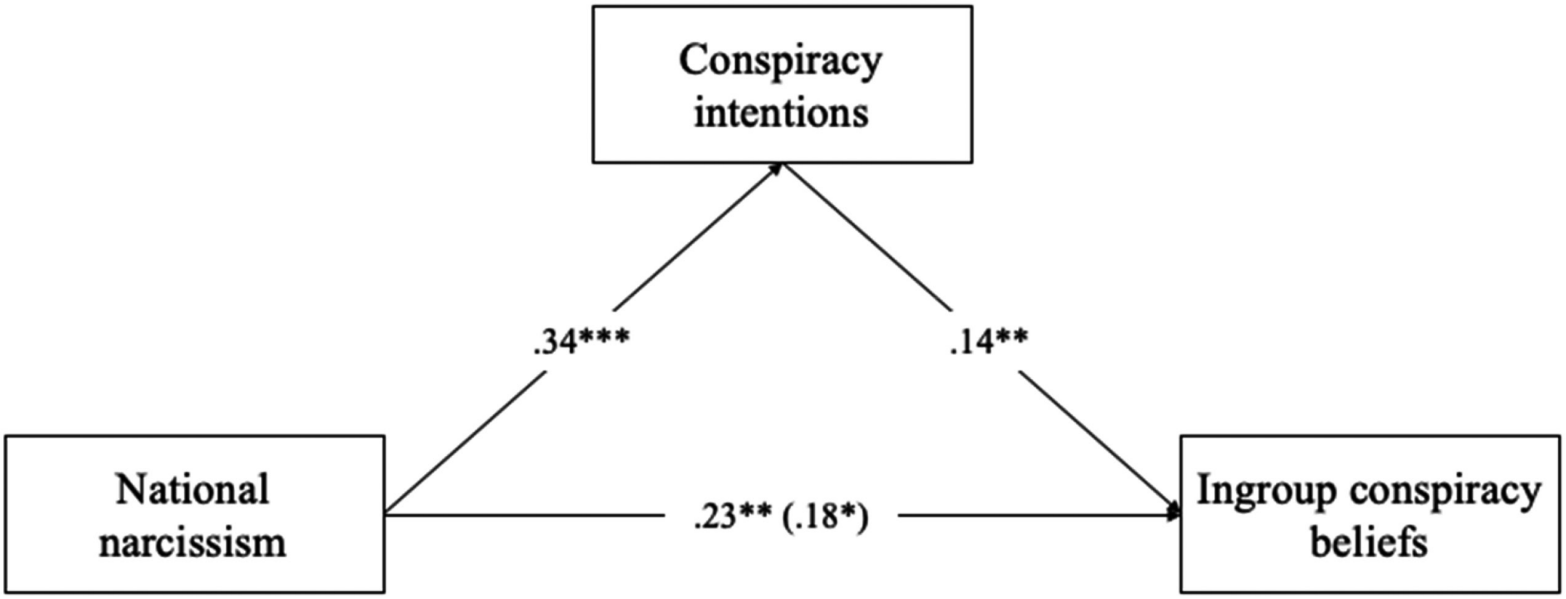
Indirect relationship between national narcissism and conspiracy beliefs via intentions (Study 3). *p < .05. **p < .01. ***p < .001. Entries are standardized coefficients; the direct path is reported in brackets; the total effect is reported without brackets. All paths controlled for political ideology (both linear and quadratic) and national identification

We then tested the hypothesis that conspiracy intentions would account for the relationship between national narcissism and in‐group conspiracy beliefs. Using Model 4 in SPSS PROCESS, we tested an indirect path model, with national narcissism as the predictor (controlling for national identification, alongside both linear and quadratic political ideology), in‐group conspiracy beliefs as the dependent variable, and conspiracy intentions as the mediator. National narcissism significantly predicted conspiracy intentions, which, in turn, predicted in‐group conspiracy beliefs. Conspiracy intentions significantly mediated the relationship between national narcissism and conspiracy beliefs, standardized indirect relationship = .05, 95% CI [0.01, 0.10]^4^ (Figure 1).

In line with our pre‐registration, we conducted exploratory analyses to examine the effect of being a Trump (vs. Clinton) voter. Intentions to conspire against the national in‐group were significantly higher among Trump (vs. Clinton) voters, *t*(418.58) = 3.74, *p* < .001. Furthermore, Trump (vs. Clinton) voters were significantly higher in national identification, *t*(447.94) = 13.50, *p* < .001, and national narcissism *t*(439.27) = 17.12, *p* < .001. However, there was no significant difference in in‐group conspiracy beliefs between voters, *t*(469) = 0.30, *p* = .77. Controlling for Trump (vs. Clinton) vote did not change the pattern of results of our main regression models (see Appendix S1).

### Discussion

Study 3 offered further support for our hypotheses. Even though our measure included items that would blatantly refer to engaging in secretly harming fellow citizens, US national narcissism (but not identification) significantly predicted intentions to engage in such governmental conspiracies. Interestingly, voting for Trump (vs. Clinton) was also associated with higher conspiracy intentions (although not in‐group conspiracy beliefs).

In line with our hypothesis, conspiracy intentions mediated the relationship between national narcissism and in‐group conspiracy beliefs. However, contrary to our expectations, national narcissism still positively predicted in‐group conspiracy beliefs even when we accounted for conspiracy intentions. This contradicts past research by Cichocka, Marchlewska, Golec de Zavala and colleagues (2016), who found that US national narcissism was not associated with conspiracy beliefs about the in‐group, but is consistent with more recent research by Golec de Zavala and Federico (2018), who found that national narcissism was associated with generalized belief in conspiracies, especially after the 2016 Trump election (see also Bertin et al., 2021). Arguably, the highly polarized context of the Trump presidency might have also intensified the association between national narcissism and in‐group conspiracy beliefs (see Kofta & Sedek, 2005). As Study 3 was correlational, it did not allow us to establish the causal association between conspiracy intentions and beliefs. Thus, it is possible that suspecting other in‐group members of conspiring would also further predict intentions to engage in conspiracies.^5^

## STUDY 4

Study 3 indicated that a willingness to conspire against fellow in‐group members might explain the link between collective narcissism and conspiracy beliefs about the in‐group. Interestingly, we found that willingness to conspire was stronger among Trump (vs. Clinton) voters. Populists, like Trump, are thought to assert who the ‘real’ members of society are, showing hostility towards those who do not fit into that category (Müller, 2016). Thus, on the one hand, one could argue that those high in collective narcissism might only be willing to conspire against those in their in‐group that they do not perceive as typical members. On the other hand, those high in collective narcissism may be even more prone to conspiring against typical in‐group members, as these members may be less suspicious towards them (Foddy et al., 2009) and less sensitive to potential in‐group threat. To investigate these possibilities, Study 4 measured the extent to which those willing to conspire perceived the targets of their conspiring as typical of the in‐group.

Furthermore, Study 4 sought to examine whether the relationship between collective narcissism and conspiracy intentions can be accounted for by other variables that may predict general willingness to conspire. For example, as shown by Douglas and Sutton (2011), conspiracy intentions are related to Machiavellianism. Therefore, Study 4 accounted for Machiavellianism, alongside the other Dark Triad personality traits of psychopathy and individual narcissism (Paulhus & Williams, 2002). Moreover, several intention items we have relied on so far have focused on conspiratorial surveillance. Past work has shown that support for government authority to engage in surveillance and censorship (e.g. Cohrs et al., 2005; Zhai et al., 2022) is linked to social dominance orientation (SDO; Pratto et al., 1994). Thus, we checked whether SDO might also play a role in the willingness to engage in surveillance conspiracies. Theoretically, SDO may also be linked to a general willingness to conspire as an attempt to protect against group members perceived as threatening to the in‐group's competitive status or dominance (see Perry et al., 2013). Consequently, we tested whether national narcissism remains a robust predictor of conspiracy intentions when controlling for the Dark Triad and SDO. The hypotheses, design and analyses were pre‐registered.

### Method

#### Participants and design

Our a priori power analysis was conducted to account for the interaction between national narcissism and perceived in‐group typicality. G*Power indicated that to detect a knockout interaction using the effect size of *r* = .26^6^ with a power of .80, a minimum sample size of 444 was required. To account for possible order effects, we planned to collect double this required sample size (see the pre‐registration for details). The survey was completed by 1064 Polish participants, 516 men, 548 women, aged 18–83 (*M*_age_ = 47.07, *SD*_age_ = 16.31). Roughly half completed the perceived in‐group typicality items before the conspiracy intentions items (*N* = 539), and the other half completed the conspiracy intentions items before the perceived in‐group typicality items (*N* = 525).

Participants completed the measures of national narcissism, identification, SDO and Dark Triad personality traits in randomized order. Then, they were presented with the perceived in‐group typicality and conspiracy intentions items (counterbalanced). Finally, participants reported their political ideology, voting intentions and demographics (age, gender and education). Unless noted otherwise, we used response scales from *definitely no(t)* (1) to *definitely yes* (7).

#### Measures

*National Narcissism* (*M* = 4.21, *SD* = 1.71, *α* = .94) and *National Identification* (*M* = 5.32, *SD* = 1.40, *α* = .89) were measured as in Study 1.

*Social Dominance Orientation* (*M* = 2.60, *SD* = 1.04, *α* = .64) was measured using Pratto and colleagues' (2013) Polish version of the four‐item Short Social Dominance Orientation scale (e.g. ‘Group equality should be our ideal’ [reverse‐coded]).

*Dark Triad Personality Traits* were measured using Czarna and colleagues' (2016) Polish translation of the 12‐item Dirty Dozen scale, capturing individual narcissism (*M* = 2.88, *SD* = 1.37, *α* = .84; e.g. ‘I tend to seek prestige or status’), psychopathy (*M* = 2.47, *SD* = 1.22, *α* = .76; e.g. ‘I tend to lack remorse’) and Machiavellianism (*M* = 2.23, *SD* = 1.29, *α* = .88; e.g. ‘I tend to manipulate others to get my way’).

*Conspiracy Intentions* (*M* = 2.37, *SD* = 1.49, *α* = .86) were measured similarly to Study 1, with three items asking participants how likely they would be to engage in secret wiretapping, the monitoring of web activity, and spreading false information about fellow Poles.

*Perceived In‐group Typicality* (*M* = 3.41, *SD* = 1.50, *α* = .88) was measured with three items asking participants how similar to typical Poles they believe citizens who are wiretapped, have their web activity monitored, and have false information spread about them by the Polish secret service to be, using a response scale from *not at all similar* (1) to *very similar* (7).

*Political Ideology* (*M* = 3.98, *SD* = 1.59) was measured as in Study 1.

*Voting Intentions* were measured by asking participants ‘If parliamentary elections were held in Poland today, what party/election committee would you be willing to vote for?’ Participants indicated their response from a list of parties (the national populist party in power, *Law and Justice N* = 208, coded as 1 versus other *N* = 856, coded as 0; see Appendix S1 for all options presented).

*Demographics*. Age, gender and education (*M* = 2.75, *SD* = 0.90) were measured as in Study 1.

### Results

As hypothesized, Polish national narcissism (but not identification) had a significant positive correlation with conspiracy intentions (Table 7). Furthermore, right‐wing ideology, perceived in‐group typicality, SDO, individual narcissism, psychopathy and Machiavellianism all had positive significant relationships with conspiracy intentions.

**TABLE 7:** Zero‐order correlations (Study 4)

	1	2	3	4	5	6	7	8	9
1. Conspiracy intentions	–	.23***	.04	.22***	.39***	.28***	.35***	.18***	.38***
2. National narcissism		–	.63***	.01	.07*	.21***	.01	.46***	.09**
3. National identification			–	−.15***	−.13***	.07*	−.19***	.38***	.05
4. SDO				–	.29***	.18***	.30***	.07*	.07*
5. Machiavellianism					–	.64***	.72***	.05	.20***
6. Individual narcissism						–	.47***	.06*	.16***
7. Psychopathy							–	.03	.20***
8. Political ideology								–	.08**
9. Perceived in‐group typicality									–

*p < .05. **p < .01. ***p < .001.

Next, in line with our pre‐registration, we constructed a regression model with national narcissism and identification as predictors, and conspiracy intentions as the dependent variable using the *lavaan* R package. As hypothesized, conspiracy intentions were positively predicted by national narcissism, *B* = 0.29, 95% CI [0.22, 0.35], *β* = .33, *p* < .001, and negatively predicted by national identification, *B* = −0.18, 95% CI [−0.26, −0.10], *β* = −.17, *p* < .001; *F*(2, 1061) = 38.68, *p* < .001, *R* ^*2*^_*adj*_ = .07.

We then proceeded to our pre‐registered exploratory analyses. We conducted regression analyses with national narcissism, identification, SDO, Machiavellianism, narcissism, and psychopathy as predictors, and conspiracy intentions as the dependent variable. In this model, the relationship between national narcissism (but not identification) and conspiracy intentions remained positive and significant (Table 8). Furthermore, this relationship even remained significant when additionally controlling for political orientation (political ideology, linear and quadratic, voting intentions) and demographic variables (age, gender and education; see Table 8). Of the co‐variates, conspiracy intentions were positively predicted by SDO, Machiavellianism, psychopathy and voting intentions and negatively predicted by age.

**TABLE 8:** Regression model with conspiracy intentions as the dependent variable (Study 4)

Variables	*B* [95% CI]	*β*	*p*	*B* [95% CI]	*β*	*p*
National narcissism	0.20 [0.14, 0.26]	.23	<.001	0.14 [0.07, 0.21]	.16	<.001
National identification	−0.03 [−0.11, 0.05]	−.03	.456	−0.03 [−0.10, 0.05]	−.02	.524
SDO	0.14 [0.06, 0.22]	.10	.001	0.12 [0.04, 0.21]	.09	.003
Machiavellianism	0.29 [0.19, 0.39]	.25	<.001	0.26 [0.16, 0.37]	.23	<.001
Individual narcissism	−0.02 [−0.09, 0.06]	−.02	.674	−0.01 [−0.08, 0.07]	−.01	.909
Psychopathy	0.18 [0.08, 0.27]	.14	<.001	0.18 [0.09, 0.28]	.15	<.001
Political ideology				0.04 [−0.03, 0.10]	.04	.267
Political ideology^2^				−0.01 [−0.03, 0.02]	−.01	.632
Voting intentions				0.37 [0.12, 0.62]	.10	.003
Age				−0.01 [−0.01, 0.01]	−.06	.046
Gender (0 = Male, 1 = Female)				0.01 [−0.16, 0.16]	.01	.993
Education				−0.06 [−0.15, 0.03]	−.04	.170
*F, R* ^*2*^_*adj*_	*F*(6, 1057) = 48.34, *p* < .001, *R* ^*2*^_*adj*_ = .21			*F*(12, 1051) = 26.32, *p* < .001, *R* ^*2*^_*adj*_ = .22		

Finally, to investigate the way in which those willing to conspire perceive the in‐group members they are targeting, we conducted a regression analysis with national narcissism (mean‐centred), identification, perceived in‐group typicality (mean‐centred) and an interaction term between national narcissism and perceived in‐group typicality as the predictors, and conspiracy intentions as the dependent variable, *F*(4, 1059) = 71.93, *p* < .001, *R* ^*2*^_*adj*_ = .21. This model revealed that conspiracy intentions were positively predicted by national narcissism, *B* = 0.25, 95% CI [0.19, 0.31], *β* = .29, *p* < .001, perceived in‐group typicality, *B* = 0.36, 95% CI [0.30, 0.41], *β* = .36, *p* < .001, and the interaction between national narcissism and perceived in‐group typicality, *B* = 0.07, 95% CI [0.04, 0.09], *β* = .13, *p* < .001, and negatively predicted by national identification, *B* = −0.16, 95% CI [−0.23, −0.09], *β* = −.15, *p* < .001. When controlling for all other variables measured, all coefficients except the one for national identification remained significant (see Appendix S1).

**FIGURE 2:**
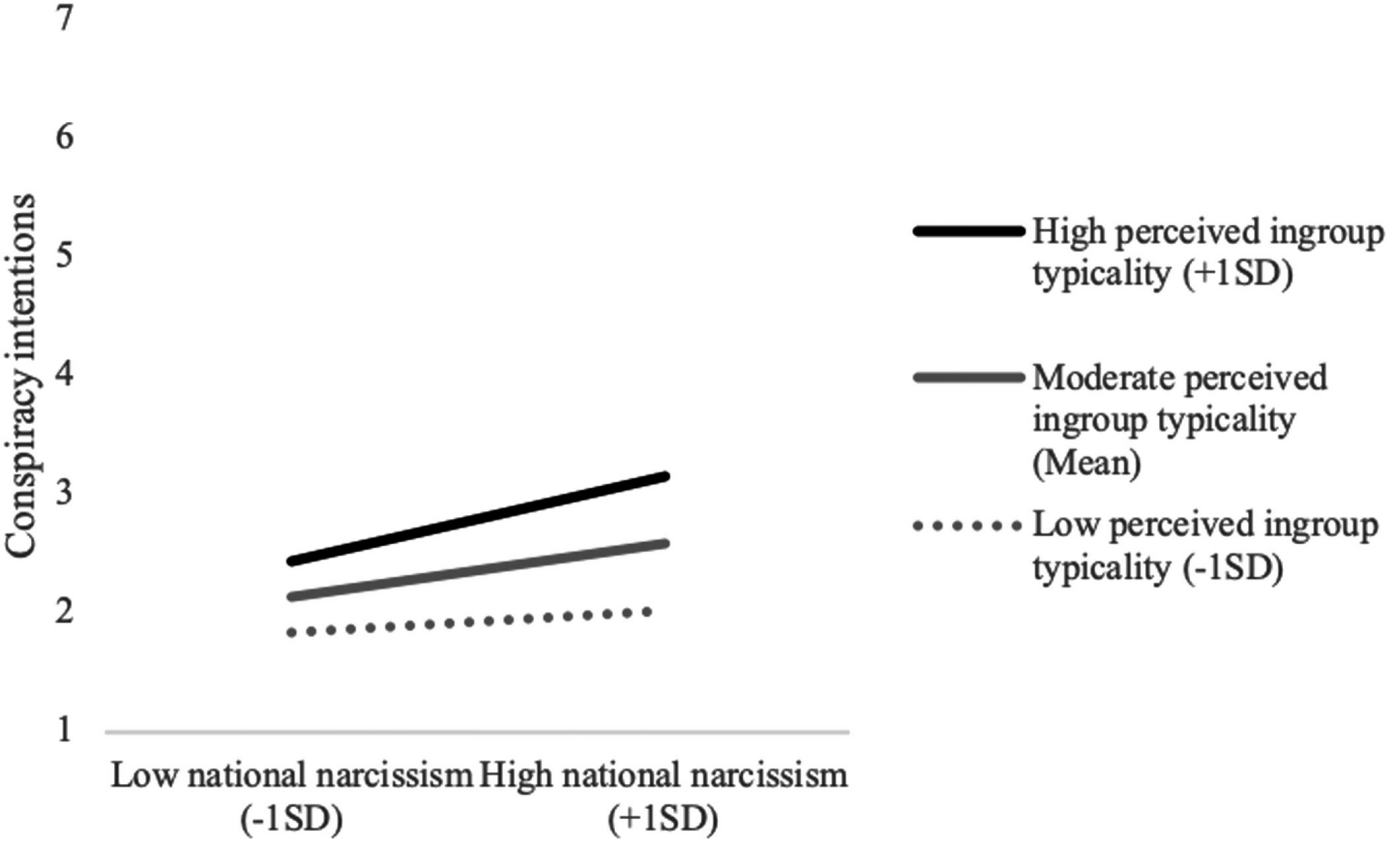
Simple slopes plot for the moderating effect of perceived in‐group typicality on the relationship between national narcissism and conspiracy intentions (Study 4)

To inspect the nature of the moderating effect of perceived in‐group typicality on the link between national narcissism and conspiracy intentions, we conducted a simple slopes analysis, which revealed that the link between national narcissism and conspiracy intentions was only positive and significant at moderate, *B* = 0.04, 95% CI [0.06, 0.21], *β* = .13, *p* < .001, and high levels of perceived in‐group typicality, *B* = 0.21, 95% CI [0.11, 0.31], *β* = .21, *p* < .001, but not when perceived in‐group typicality was low, *B* = 0.05, 95% CI [−0.02, 0.13], *β* = .05, *p* = .153 (see Figure 2).^7^

## DISCUSSION

Study 4 again replicated the link between collective narcissism and conspiracy intentions. This relationship was especially strong when the targets of the conspiracies were perceived as at least moderately typical of the in‐group, suggesting that those scoring high in national narcissism were only willing to conspire against those perceived as genuine members of their nation (rather than those who might be sub‐typed as members of other groups). Interestingly, if these group members were also perceived as similar to the self, this could further strengthen the tendency to project one's malevolent intentions onto them (Ames, 2004). Collective narcissists might also view typical, yet disloyal in‐group members as the most threatening to the in‐group image, making them likely targets of conspiracies.

We further demonstrated that national narcissism is a unique predictor of conspiracy intentions, over and above any effects of political and personality factors. The effect of national narcissism was observed in parallel with the significant effects of Machiavellianism (replicating Douglas & Sutton, 2011), psychopathy, SDO and voting intentions (with higher conspiracy intentions reported by those voting for the ruling nationalist populist Law and Justice party compared with other voters), and when accounting for individual narcissism. These findings complement past research by Enders and colleagues (2021), showing that conspiracy beliefs can be organized by anti‐social orientations (e.g. Machiavellianism) or identity concerns (e.g. partisanship; see also Uscinski et al., 2021). Our work suggests that these two dimensions might also come into play when explaining conspiracy intentions.

## INTERNAL META‐ANALYSES

In order to gain an overall understanding of our findings, we meta‐analysed the zero‐order correlations^8^ between: 1) collective narcissism and conspiracy intentions, 2) in‐group identification and conspiracy intentions, and 3) right‐wing ideology and conspiracy intentions across the four main studies (see the Appendix S1 for results including the Pilot). To carry this out, we tested three separate Robust Variance Estimation (RVE) models using the *robumeta* R package (Fisher et al., 2017). Due to the small number of studies included in these respective meta‐analyses, the significance level was adjusted to a more conservative *p* < .01 to correct for the low degrees of freedom (*df* < 4; see Tipton & Pustejovsky, 2015). The *JASP* R skin (JASP Team, 2020) was used to obtain our Bayes Factors, applying the recommended Half Cauchy τ prior (*SD* = 0.5; see Harrer et al., 2019). Below, we report the key results, while full results are presented in the Appendix S1.

### Results

**FIGURE 3:**
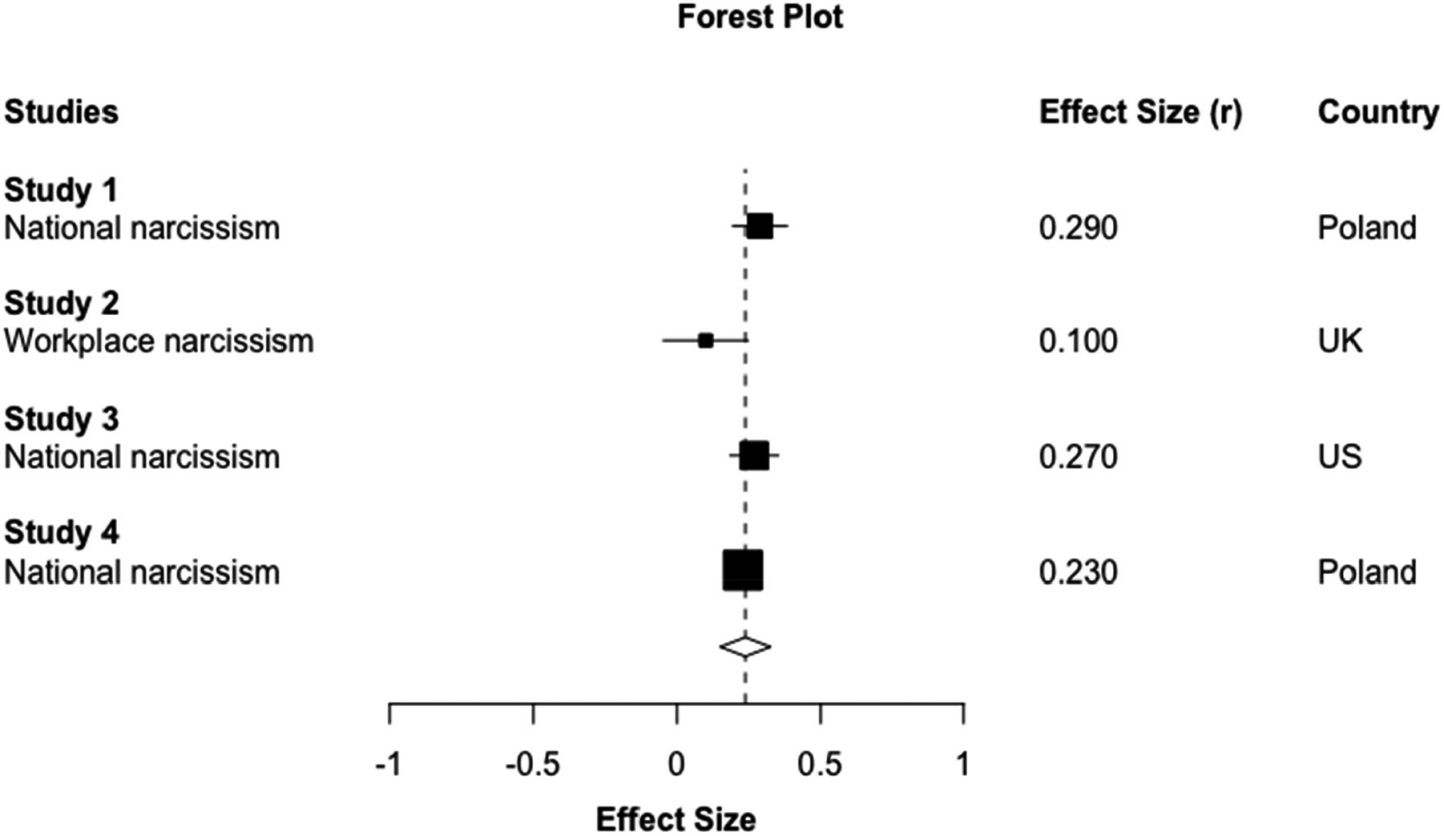
Forest plot of the collective narcissism effect sizes

Collective narcissism had a small significant positive meta‐analytic association with conspiracy intentions, *r* = .24, 95% CI [.15, .33], *t*(2.59) = 9.48, *p* = .004 (see Figure 3), and the Bayes Factor provided weak evidence for its directional hypothesis, BF_10_ = 3.48. In‐group identification did not have a significant meta‐analytic association with conspiracy intentions, *r* = .04, 95% CI [−.16, .24], *t*(2.94) = 0.59, *p* = .598, and the Bayes Factor provided moderate‐to‐strong evidence for its null hypothesis, BF_10_ = 0.08. Finally, right‐wing ideology did not have a significant meta‐analytic association with conspiracy intentions, *r* = .18, 95% CI [.06, .31], *t*(1.87) = 6.85, *p* = .025, as it did not pass the adjusted *p* < .01 significance level, and the Bayes Factor provided uncertain evidence for its directional hypothesis, BF_10_ = 1.12.

### Discussion

The results of our internal meta‐analyses provided further evidence that collective narcissism, but not in‐group identification, is associated with intentions to conspire against fellow in‐group members. The effect size obtained for collective narcissism is similar to that of the meta‐analytic association between defensive in‐group identity (mostly opersationalised as collective narcisssism) and conspiracy beliefs (*r* = .20; Biddlestone et al., 2022). The respective Bayes Factors confirmed that the collective narcissism directional hypothesis was more likely than the null, and the in‐group identification null hypothesis was more likely than the directional. Importantly, our findings in the workplace context (Study 2) were weaker than those in the political context. Although the current number of studies was too small to conduct moderation analyses, future work should examine the degree to which context matters in explaining these processes.

A non‐significant meta‐analytic effect size was obtained for right‐wing ideology, but this may have been due to too few studies, as indicated by the uncertain evidence for the directional hypothesis provided by its Bayes Factor. Our inclusion of single‐item measures of ideological self‐placement warrants caution when drawing conclusions around our findings regarding this variable, especially considering the cross‐cultural differences in the underpinnings of the left–right political orientation (e.g. Wojcik et al., 2021).

## GENERAL DISCUSSION

Across four studies (and one pilot), we examined how identity processes might be linked to support for conspiratorial actions against the in‐group. While traditional accounts of in‐group identity might suggest that people are willing to act against outgroups rather than in‐groups (e.g. Hewstone et al., 2002; cf. Castano et al., 2002; Marques & Paez, 1994), we show that this might not be true for certain forms of in‐group identity. We found that collective narcissism was associated with intentions to conspire against in‐group members, both in the national and workplace (albeit less strongly) contexts. This relationship was robust across three countries: Poland (Pilot Study, Studies 1 and 4), the United Kingdom (Study 2) and the United States (Study 3).

Collective narcissism compensates for frustrated needs (Cichocka et al., 2018; Golec de Zavala et al., 2019). Thus, for those scoring high in collective narcissism, it is the group that serves the individual, rather than the individual who serves the group (Cichocka, 2016). As those scoring high in collective narcissism are more firmly invested in the in‐group, they should be more likely to try to satisfy their needs via taking advantage of the group. This implies that those high in collective narcissism might be ready to sacrifice in‐group members and collude against them, if this helps them further their agendas (see Douglas & Sutton, 2011). These conspiratorial efforts seem directed towards in‐group members perceived as typical of the in‐group. Our findings add to the growing literature showing that collective narcissism does not only predict hostile outgroup attitudes, but that it is also linked to problematic relations within the in‐group (Cichocka et al., 2021; Cichocka & Cislak, 2020; Gronfeldt et al., 2022; Marchlewska et al., 2020).

Despite collective narcissism often stemming from a frustration of *personal* needs (such as low personal control; see Bertin et al., 2022; Cichocka et al., 2018), Study 4 demonstrated that intentions to conspire against the in‐group can be uniquely accounted for by collective narcissism over and above any effects of personality predictors. Thus, while a general readiness to conspire against others may be accounted for by a personal lack of empathy (i.e. psychopathy) and tendency to treat other individuals instrumentally (i.e. Machiavellianism), defensive in‐group identity still uniquely explains these processes at the collective self level. The relationship between collective narcissism and conspiracy intentions remained significant even when adjusting for SDO – a known predictor of support for government surveillance (e.g. Cohrs et al., 2005; Zhai et al., 2022).

This apparent dual pathway from both individual and collective self‐factors to conspiracy intentions is in line with a recent framework analysing the associations between conspiracy beliefs and different levels of self‐definition (see Brewer & Gardner, 1996). Specifically, Biddlestone and colleagues (2021) argue that conspiracy beliefs are partially explained by motives that aim to satisfy personal needs associated with the individual self (e.g. individual narcissism), interpersonal needs associated with the relational self (e.g. social exclusion) and intergroup needs associated with the collective self (e.g. collective narcissism). Importantly, these processes are likely to act dynamically and in tandem with one another (see also Cichocka, Marchlewska, & Golec de Zavala, 2016). The current work suggests that these processes might also work in parallel when predicting conspiracy intentions, although there might be differences in specific personality factors associated with belief versus willingness to engage in conspiracies.

Their personal willingness to conspire can also explain why collective narcissists watch out for conspiracies within their ranks: they may perceive other in‐group members as a threat due to a projection of their own mental states. As shown by Douglas & Sutton (2011), those who are willing to conspire believe that others would do so as well. Indeed, in Study 3, conspiracy intentions partially explained the positive association between collective narcissism and belief in in‐group conspiracies, replicating Douglas & Sutton (2011) in an in‐group context. While these beliefs may involve conspiracist suspicions of others in general, this finding further demonstrates the lack of trust that collective narcissists may hold even towards their own in‐group.

Importantly, given the correlational nature of our studies, causality was not established. It is then also possible that in‐group conspiracy beliefs affected conspiracy intentions. For example, intentions to engage in conspiracies within one's group might be a response to a conviction that malevolent forces operate within one's society. Such beliefs and intentions might in fact form a positive feedback loop, which fuels a culture of intragroup suspicion and paranoia, making conspiracy narratives about the in‐group more believable and further frustrating personal needs (see also Douglas et al., 2017). This also implies that the conspiracies those high in collective narcissism appear willing to engage in are unlikely to satisfy the frustrated personal needs they purport to serve.

As suggested by the meta‐analysis, in‐group identification was unrelated to conspiracy intentions. However, in Studies 1 and 2 (and 4 until controlling for additional variables) we found that in‐group identification was negatively related to conspiracy intentions, once its overlap with collective narcissism was accounted for (see Cichocka, 2016). These findings suggest that more secure, non‐narcissistic in‐group identity might limit one's willingness to engage in actions that harm in‐group members. This is consistent with past work showing positive effects of identifying with groups (e.g. Randsley de Moura et al., 2009; Jetten et al., 2012; van Zomeren et al., 2008). We did not observe similar significant relationships for in‐group identification in Study 3 (and 4 when controlling for additional variables). These discrepant findings could be due to different identification measures used in different studies (Cameron, 2004; Leach et al., 2008). Another possibility is that our measures of intentions might have captured a protective willingness to dissent from the in‐group, alongside the readiness to engage in dubious, secretive plots. Indeed, members that report a positive in‐group identification may sometimes feel urged to resist conformity when the group is not living up to its own standards and values (Packer, 2007).

In Studies 3 and 4, we also explored the role of political ideology and voter preference in conspiracy intentions. Although we did not consistently find that right‐wing ideology was associated with conspiracy intentions in these two studies (and according to the main meta‐analysis, right‐wing ideology was overall unrelated to conspiracy intentions), we did find Trump voters to be higher in national narcissism and intentions to conspire than Clinton voters. Similarly, Law and Justice (vs. other) voters were more likely to report conspiracy intentions. These results shed light on why populists might be more at risk to engage in conspiracies. They tend to be convinced that elites are unanimously greedy and untrustworthy (Castanho Silva et al., 2017; Eberl et al., 2020; Stecula & Pickup, 2021), instead believing that they themselves hold a morally superior vision of what is good or bad for the nation (Müller, 2016; see also Bocian et al., 2021). This conviction may justify their willingness to engage in conspiracies that could undermine the freedoms and well‐being of fellow group members. Populist movements both in Poland and the United States appear to embody conspiratorial notions (Marchlewska et al., 2018) and, in fact, leaders may sometimes use conspiracy theories strategically (Douglas et al., 2020). Future research could examine the role of populist rhetoric in inspiring covert actions that seek to undermine in‐group cohesion and democratic principles.

Another potentially fruitful research line could examine whether conspiracy intentions might be linked to other forms of defensive (and secure) in‐group identity. For example, measuring blind patriotism (Schatz et al., 1999) or national glorification (Roccas et al., 2006) alongside collective narcissism could help uncover whether the perception of in‐group greatness as undervalued (a unique characteristic of collective narcissism) is the key process responsible for the associations we observed here. Moreover, future studies could examine whether collective narcissism, or other forms of defensive identity, might be related to conspiracy intentions against outgroups as well. Overall, identifying factors behind intentions to conspire against outgroup versus in‐group members would help elucidate the motivational underpinnings of conspiracy intentions and thus would represent an important step in developing the theoretical model of engagement in conspiracies.

## CONCLUSION

History is rich with examples of those who proclaim their commitment to their groups but end up plotting against them. Here, we examined the concomitants of personal willingness to engage in conspiracies against fellow group members. Despite their alleged in‐group love, those scoring high in collective narcissism might be ready to conspire against those perceived as most typical of the in‐group. Even though collective narcissists seem to always be on the lookout for others threatening their group, eventually they might end up being their own group's worst enemies. In the (in)famous words of George W. Bush: ‘Our enemies are innovative and resourceful, and so are we. They never stop thinking about new ways to harm our country and our people, and neither do we’ (cited in Time, 2009).

## CONFLICT OF INTEREST

None.

## AUTHOR CONTRIBUTIONS

**Mikey Biddlestone:** Conceptualization; Data curation; Formal analysis; Investigation; Methodology; Project administration; Visualization; Writing – original draft; Writing – review & editing. **Aleksandra Cichocka:** Conceptualization; Data curation; Formal analysis; Funding acquisition; Investigation; Methodology; Project administration; Supervision; Visualization; Writing – original draft; Writing – review & editing. **Michał Główczewski:** Data curation; Formal analysis; Methodology; Project administration. **Aleksandra Cislak:** Conceptualization; Data curation; Funding acquisition; Supervision; Writing – review & editing.

## ETHICS STATEMENT

Participant consent and full ethical approval in accordance with the APA Code of Conduct was obtained for all studies.

## Supporting information


Appendix S1

## Data Availability

https://osf.io/gxytw/.

## Footnotes

^1^
All significance levels and confidence intervals are from bootstrapped coefficients with 1000 resamples (*B*), standardized beta coefficients are not bootstrapped.

^2^
Study 1 additionally included measures of support for environmental and health‐related policies for purposes of different projects (Cislak, Marchlewska et al., 2021).

^3^
We also measured education, but the item included an error. Nevertheless, controlling for education did not affect the pattern of results.

^4^
This was contrary to our pre‐registered suppression hypothesis, in which we expected national narcissism to be negatively associated with conspiracy beliefs directly and positively associated with conspiracy beliefs indirectly via conspiracy intentions. Instead, although the correlation between national narcissism and in‐group conspiracy beliefs was non‐significant, our regression analyses, which controlled for in‐group identification, revealed that national narcissism positively predicted conspiracy beliefs regardless of intentions being accounted for.

^5^
Note, however, that the zero‐order correlation between collective narcissism and in‐group conspiracy beliefs was non‐significant until the overlap in variance with other variables was accounted for. Furthermore, the alternative indirect relationship between collective narcissism and conspiracy intentions through conspiracy beliefs about the in‐group was weaker than that of the hypothesized path, standardized indirect relationship = .03, 95% CI [.01, .06].

^6^
This was obtained from the meta‐analytic association between collective narcissism and support for conspiratorial actions for the pilot and three main studies so far.

^7^
We also conducted an additional regression analysis controlling for the order in which participants were presented with the conspiracy intentions and perceived in‐group typicality items (1 = conspiracy items first and 2 = typicality items first). While our hypothesized relationships were not influenced by the inclusion of this variable, conspiracy intentions were significantly stronger when participants were presented with the typicality items first (see the Appendix S1 for further details).

^8^
Although our hypotheses were based on the inclusion of overlapping variances between both conventional and narcissistic identity, recent meta‐analytic simulations urge caution over the use of standardized beta coefficients in meta‐analyses due to considerable bias (Roth et al., 2018). Therefore, we chose to analyse zero‐order correlations instead.
