# Supplementary material for: Their own worst enemy? Collective narcissists are willing to conspire against their in‐group
Source: Br J Psychol. 2022 May 6;113(4):894–916. doi: 10.1111/bjop.12569 (PMC9790724; doi:10.1111/bjop.12569)
Supplement: Supplementary file 1 — Appendix S1 [file BJOP-113-894-s001.docx]

**Supplemental Information**

**Pilot Study**

In the Pilot Study, we sought to establish whether collective narcissism would be linked to tolerance of conspiracies against the ingroup from within the group. To this end, we relied on a real-life example. We conducted the study in Poland, using the context of the government’s alleged secret purchasing of the *Pegasus* surveillance technology (Reuters, 2022). We asked participants how supportive they would be of the government using this software against its own citizens. We also controlled for support for the ruling party.

***Method***

**Participants and Design.** The study was conducted among 578 Polish participants (387 women), recruited through the internet panel *Ariadna* (*M_age_*=38.14; *SD_age_*=12.33). Sensitivity analysis with G*Power (assuming alpha of .05, power of .80, two-tailed) suggested that we had enough power to detect a small effect size of *r* = .12. National narcissism and identification (counterbalanced) were measured as predictors of support for the secret use of the *Pegasus* software by the Polish government. Participants were also asked for basic demographics (highest level of education, gender, age, and place of residence, political ideology, and the party they voted for during the last parliamentary elections).^[[1]](#footnote-1)^

**Measures**

***National Narcissism*** was measured with five items of the Collective Narcissism Scale (e.g., “If Poles had a major say in the world, the world would be a much better place”; Golec de Zavala et al., 2009), with a response scale from *definitely not* (1) to *definitely yes* (5).

***National Identification*** was measured with five items, e.g., “I feel strong ties to other Polish people” based on Cameron (2004), on a scale from *definitely not* (1) to *definitely yes* (5).

***Support for the Use of the Pegasus Software*** by the Polish government was measured with three original items: “I think that the Polish intelligence should have the "Pegasus" software”, “Polish state needs systems such as "Pegasus" even at the cost of violating the privacy of citizens”, and “If the information about the purchase of the “Pegasus” system by the Central Anticorruption Bureau were confirmed by the Polish government, I would support such a decision of Polish services”, on a scale from *definitely not* (1) to *definitely yes* (5).

***Political Ideology*** was measured with a single item asking participants to indicate their political orientation on a scale from *definitely left-wing* (1) to *definitely right-wing* (7).

***Results and Discussion***

Analysis of correlations revealed that all main variables significantly correlated with each other (Table S1).

Next, a regression analysis revealed that national narcissism (but not national identification) significantly predicted support for the use of the surveillance software, *F*(4, 573)=17.62, *p*<.001; *R*^2^*_adj_*=.10 (Table S2). Therefore, we established that collective narcissism might predict greater tolerance of conspiracies within the group.

In past work, support for surveillance measures has been associated with right‐wing authoritarianism (e.g., Cohrs et al., 2005), which tends to correlate with national narcissism (Golec de Zavala et al., 2009). Consequently, we controlled for political ideology (mean centred) and political ideology mean-centred squared (in order to test for any effects of political extremism). Although we did not measure right-wing authoritarianism directly, we found that right-wing political orientation (although not extremism) significantly predicted support for the surveillance software. Still, the effect of national narcissism remained significant after controlling for ideology. These effects remained similar when we controlled for all demographics.

Additional analyses also revealed that supporters of the ruling Law and Justice party (vs. other parties) were significantly higher in national narcissism, *t*(576)=8.05, *p*<.001, national identification, *t*(304.78)=6.82, *p*<.001, and support for the surveillance software*, t*(576)=7.01, *p*<.001. When we additionally controlled for Law and Justice party support in the regression models, results remained similar (Table S3).

**Table S1**

*Means, Standard Deviations, Reliabilities, and Zero-Order Correlations (Pilot Study)*

| Variable | *M* | *SD* | α | 1 | 2 | 3 | 4 |
| --- | --- | --- | --- | --- | --- | --- | --- |
| 1. Support for surveillance | 2.64 | 1.08 | .92 | - | .28^***^ | .18^***^ | .26^***^ |
| 2. National narcissism | 3.27 | 0.87 | .85 |  | - | .52^***^ | .35^***^ |
| 3. National identification | 3.80 | 0.81 | .86 |  |  | - | .23^***^ |
| 4. Political ideology | 3.92 | 1.35 | - |  |  |  | - |

*^***^p* < .001.

**Table S2**

*Regression Model with Support for Surveillance as the Dependent Variable (Pilot Study)*

| Variables | *B* [95% CI] | β | *p* |
| --- | --- | --- | --- |
| National narcissism | 0.25 [0.12, 0.37] | .20 | <.001 |
| National identification | 0.04 [-0.09, 0.16] | .03 | .617 |
| Political ideology | 0.15 [0.08, 0.22] | .19 | <.001 |
| Political ideology^2^ | 0.01 [-0.03, 0.05] | .02 | .596 |

**Table S3**

*Regression Model with Support for Surveillance as the Dependent Variable, Controlling for Law and Justice (vs. Other Parties) Support (Pilot Study)*

| Variables | *B* [95% CI] | β | *p* |
| --- | --- | --- | --- |
| National narcissism | 0.21 [0.08, 0.34] | .17 | .005 |
| National identification | 0.02 [-0.11, 0.17] | .02 | .739 |
| Political ideology | 0.10 [0.02, 0.19] | .13 | .012 |
| Political ideology^2^ | -0.01 [-0.04, 0.03] | -.01 | .806 |
| Party support (0 = other, 1 = Law and Justice) | 0.43 [0.19, 0.65] | .17 | <.001 |

**Table S4**

*Regression Model with Support for Surveillance as the Dependent Variable, Controlling for the Experimental Condition (Pilot Study)*

| Variables | *B* [95% CI] | β | *p* |
| --- | --- | --- | --- |
| National narcissism | 0.21 [0.09, 0.33] | .17 | .001 |
| National identification | 0.02 [-0.11, 0.15] | .02 | .759 |
| Political ideology | 0.10 [0.03, 0.18] | .13 | .010 |
| Political ideology^2^ | -0.01 [-0.04, 0.03] | -.01 | .813 |
| Party support (0 = other, 1 = Law and Justice) | 0.42 [0.18, 0.65] | .17 | .004 |
| Condition (0 = Self-affirmation, 1 = control) | -0.07 [-0.22, 0.09] | -.03 | .393 |

**Study 3 Additional Analyses**

**Table S5**

*Regression Model with Conspiracy Intentions and Beliefs as Dependent Variables, Controlling for Trump (vs. Clinton) Support (Study 3)*

|  | Conspiracy intentions | | | Conspiracy beliefs | | |
| --- | --- | --- | --- | --- | --- | --- |
| Variables | *B* [95% CI] | β | *p* | *B* [95% CI] | β | *p* |
| National narcissism | 0.23 [0.11, 0.34] | .32 | <.001 | 0.21 [0.05, 0.37] | .20 | .016 |
| National identification | -0.03 [-0.10, 0.05] | -.04 | .415 | -0.32 [-0.45, -0.17] | -.30 | .001 |
| Political ideology | -0.06 [-0.18, 0.06] | -.11 | .395 | -0.12 [-0.25, 0.01] | -.15 | .073 |
| Political ideology^2^ | -0.02 [-0.07, 0.03] | -.05 | .361 | -0.002 [-0.08, 0.07] | -.003 | .943 |
| Vote (0 = Clinton; 1 = Trump) | 0.10 [-0.11, 0.31] | .08 | .357 | 0.29 [-0.01, 0.58] | .14 | .045 |

**Study 4 Additional Analyses**

**Table S6**

*Regression Model Controlling for Order Effects with Conspiracy Intentions as the Dependent Variable (Study 4)*

| Variables | *B* [95% CI] | β | *p* |
| --- | --- | --- | --- |
| National narcissism | 0.13 [0.07, 0.19] | .15 | < .001 |
| National identification | -0.03 [-0.11, 0.04] | -.03 | .388 |
| Perceived ingroup typicality | 0.28 [0.23, 0.33] | .28 | < .001 |
| National narcissism X Perceived ingroup typicality | 0.05 [0.03, 0.08] | .11 | < .001 |
| Political ideology | 0.03 [-0.03, 0.09] | .03 | .358 |
| Political ideology^2^ | -0.01 [-0.03, 0.02] | -.01 | .646 |
| Voting intentions (1 = Law and Justice, 0 = Other) | 0.33 [0.10, 0.56] | .09 | .005 |
| SDO | 0.12 [0.02, 0.20] | .08 | .002 |
| Individual narcissism | -0.01 [-0.08, 0.07] | -.01 | .865 |
| Psychopathy | 0.13 [0.04, 0.22] | .11 | .004 |
| Machiavellianism | 0.22 [0.12, 0.31] | .19 | < .001 |
| Age | -0.01 [-0.01, -0.01] | -.07 | .014 |
| Gender (1 = Female, 0 = Male) | 0.04 [-0.12, 0.19] | .01 | .637 |
| Education | -0.06 [-0.14, 0.03] | -.03 | .206 |
| Order (1 = Intentions first, 2 = Typicality first) | 0.23 [0.08, 0.38] | .08 | .003 |

In line with our pre-registered exploratory analyses, we conducted separate regression analyses using the *lavaan* package in R, but this time only including participants that were presented with the conspiracy intentions (vs. typicality) items first. We found that the results thus far remained the same for this subsample, *F*(14 ,510) = 19.67, *p* < .001, *R^2^_adj_* = .33 (see Table S7).

**Table S7**

*Regression Model with all Independent Variables and Conspiracy Intentions as the Dependent Variable, Only Including the Subsample that was Presented with the Conspiracy Intentions Items Before the Perceived Ingroup Typicality Item (Study 4)*

| Variables | *B* [95% CI] | β | *p* |
| --- | --- | --- | --- |
| National narcissism | 0.15 [0.06, 0.23] | .18 | .001 |
| National identification | -0.06 [-0.16, 0.04] | -.06 | .224 |
| Perceived ingroup typicality | 0.20 [0.13, 0.26] | .22 | < .001 |
| National narcissism X Perceived ingroup typicality | 0.05 [0.01, 0.08] | .10 | .006 |
| Political ideology | 0.01 [-0.07, 0.09] | .01 | .786 |
| Political ideology^2^ | -0.01 [-0.03, 0.03] | -.01 | .876 |
| Voting intentions (1 = Law and Justice, 0 = Other) | 0.46 [0.16, 0.76] | .13 | .003 |
| SDO | 0.15 [0.05, 0.25] | .11 | .004 |
| Individual narcissism | 0.05 [-0.04, 0.14] | .05 | .312 |
| Psychopathy | 0.08 [-0.04, 0.20] | .07 | .192 |
| Machiavellianism | 0.27 [0.15, 0.40] | .25 | < .001 |
| Age | 0.01 [-0.01, 0.01] | -.01 | .924 |
| Gender (1 = Female, 0 = Male) | 0.11 [-0.09, 0.31] | .04 | .275 |
| Education | -0.03 [-0.14, 0.08] | -.02 | .590 |

Moderation analysis controlling for all variables only including the subsample that was presented with the conspiracy intentions items before the perceived ingroup typicality items also revealed that the link between collective narcissism and conspiracy intentions was only significant when group members were perceived as moderately, *B* = 0.14, 95% CI [0.05, 0.23], β = .14, *p* = .003, or highly typical of the ingroup, *B* = 0.21, 95% CI [0.07, 0.35], β = .21, *p* = .004, but not when perceived ingroup typicality was low, *B* = 0.06, 95% CI [-0.01, 0.15], β = .06, *p* = .119 (see Figure S1).

**Figure S1**

*Simple Slopes Plot for the Moderating Effect of Perceived Ingroup Typicality on the Relationship Between Collective Narcissism and Conspiracy Intentions, Only Including the Subsample that was Presented with the Conspiracy Intentions Items First (Study 4)*

In our additional pre-registered exploratory analyses, we found that perceived ingroup typicality was positively predicted by conspiracy intentions (mean-centered), *F*(14 ,510) = 7.25, *p* < .001, *R^2^_adj_* = .14 (see Table S8), but not the interaction between conspiracy intentions and national narcissism.

**Table S8**

*Regression Model with all Independent Variables and Perceived Ingroup Typicality as the Dependent Variable, Only Including the Subsample that was Presented with the Conspiracy Intentions Items Before the Perceived Ingroup Typicality Item (Study 4)*

| Variables | *B* [95% CI] | β | *p* |
| --- | --- | --- | --- |
| National narcissism | 0.01 [-0.10, 0.12] | .01 | .820 |
| National identification | 0.03 [-0.09, 0.16] | .03 | .633 |
| Conspiracy intentions | 0.30 [0.20, 0.41] | .27 | < .001 |
| National narcissism X Conspiracy intentions | 0.04 [-0.01, 0.09] | .06 | .135 |
| Political ideology | 0.08 [-0.02, 0.18] | .09 | .095 |
| Political ideology^2^ | 0.01 [-0.03, 0.05] | .03 | .538 |
| Voting intentions (1 = Law and Justice, 0 = Other) | 0.01 [-0.38, 0.41] | .01 | .946 |
| SDO | -0.03 [-0.16, 0.10] | -.02 | .686 |
| Individual narcissism | -0.02 [-0.14, 0.10] | -.02 | .737 |
| Psychopathy | 0.14 [-0.01, 0.29] | .11 | .064 |
| Machiavellianism | 0.07 [-0.10, 0.23] | .05 | .426 |
| Age | 0.01 [-0.01, 0.01] | .03 | .504 |
| Gender (1 = Female, 0 = Male) | 0.03 [-0.22, 0.29] | .01 | .810 |
| Education | -0.03 [-0.17, 0.12] | -.01 | .727 |

**Internal meta-analyses**

Once we obtained the respective meta-analytic effect sizes, we used the *brms* package (Bürkner, 2017) to obtain the between-study variances. Furthermore, we computed the evidential value and averaged estimated power levels of our significant effect sizes by using the ­*P-Curve* app 4.0 to produce *P-Curves* (Simonsohn et al., 2014)*.*

***Collective narcissism***

Figure S2 shows the Bayesian posterior predictive distribution for the collective narcissism data. This indicates that the density of the replications is roughly similar to that of the data, confirming that the model successfully converged. The effect sizes are normally distributed around the obtained effect size, with some minor kurtosis (Figure S3). The sensitivity analysis confirmed that the estimated within-study effects (*ρ*  = .80) were appropriate, and both the effect size heterogeneity, I^2^ = 41.57%, and between study variance, τ^2^ = .01, were suitable. However, the between-study variance distribution indicated some notable positive skew (see Figure S4). The *P-Curve* also indicated that the significant effects contained evidential value, *Z* = 10.02, *p* < .001, and that the average estimated power level was 99%, 95% CIs [99%, 99%] (see Figure S5).^[[2]](#footnote-2)^

**Figure S2**

*Bayesian posterior predictive distribution for collective narcissism (iterations: 6,000)*


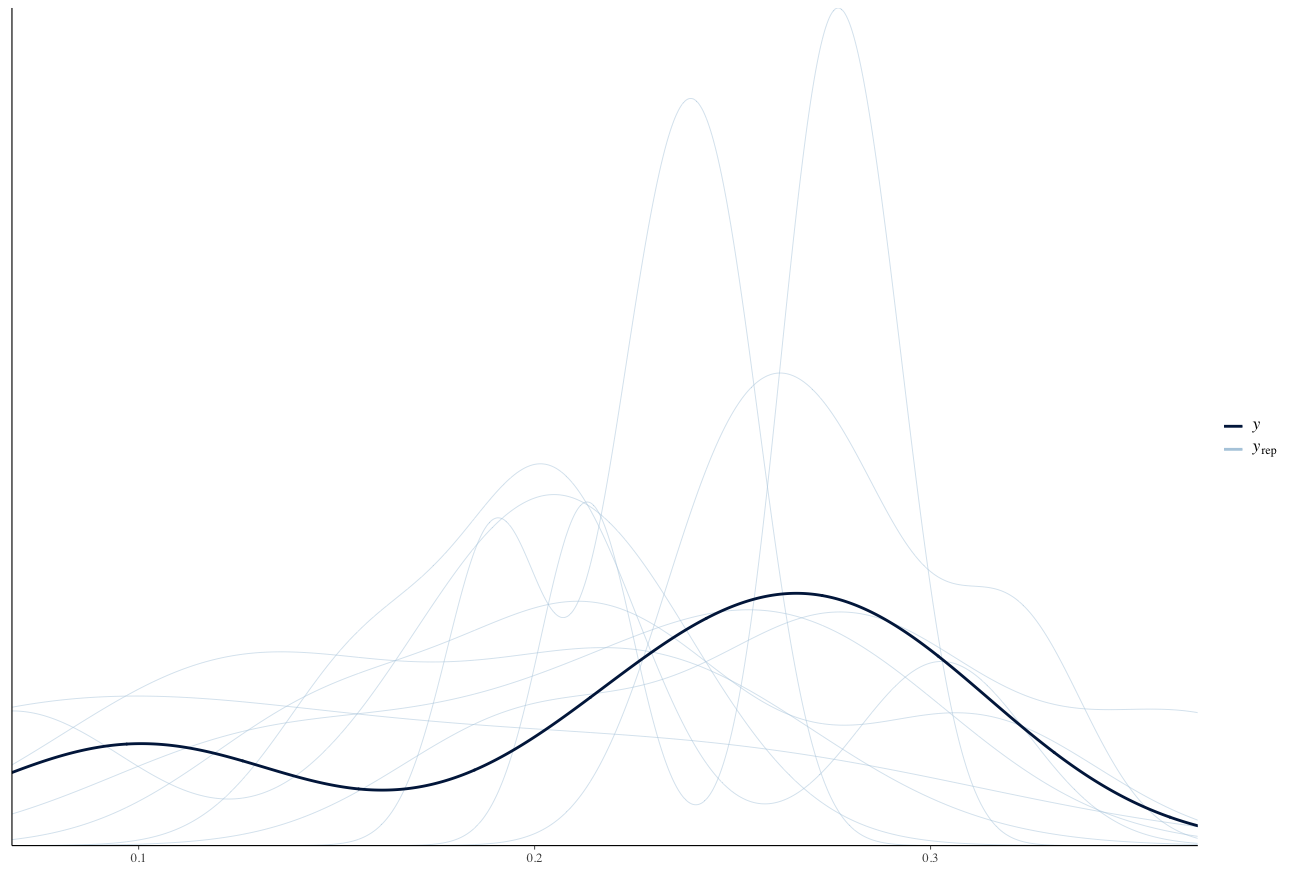


**Figure S3**

*Effect size distribution for collective narcissism* (central *r* = .23)

**
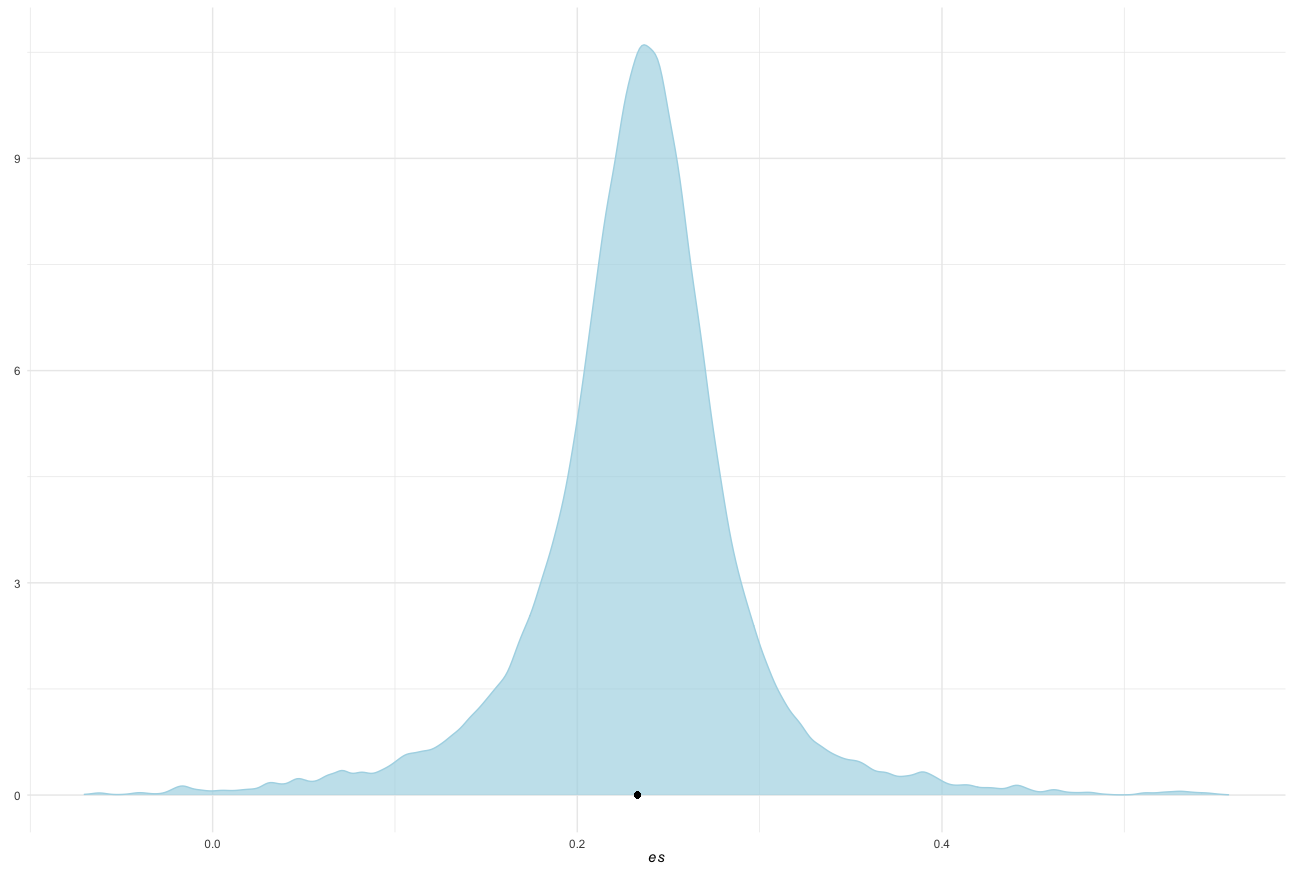
**

**Figure S4**

*Between-study variance distribution for collective narcissism* (central τ = .08)


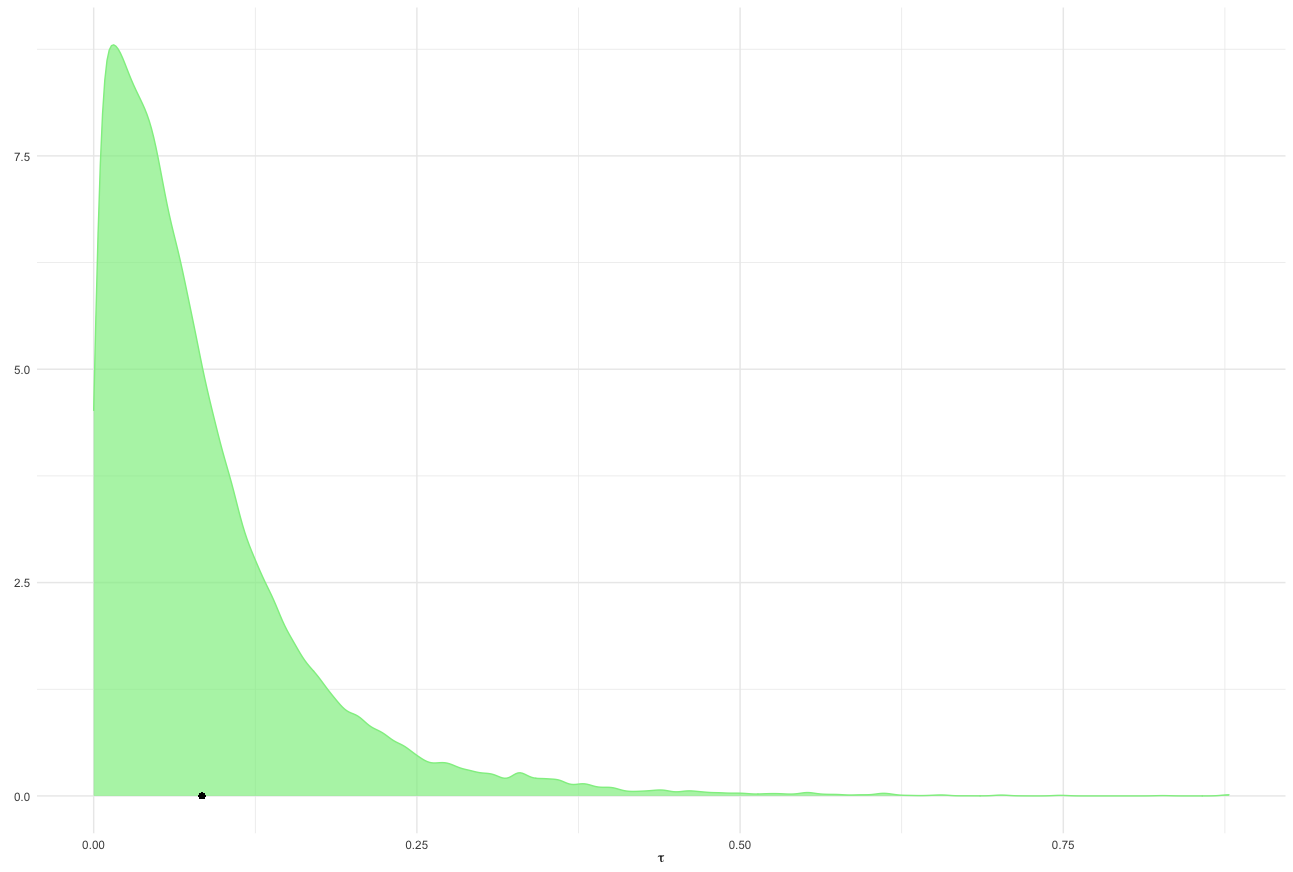


**Figure S5**

*P-Curve for collective narcissism*

*
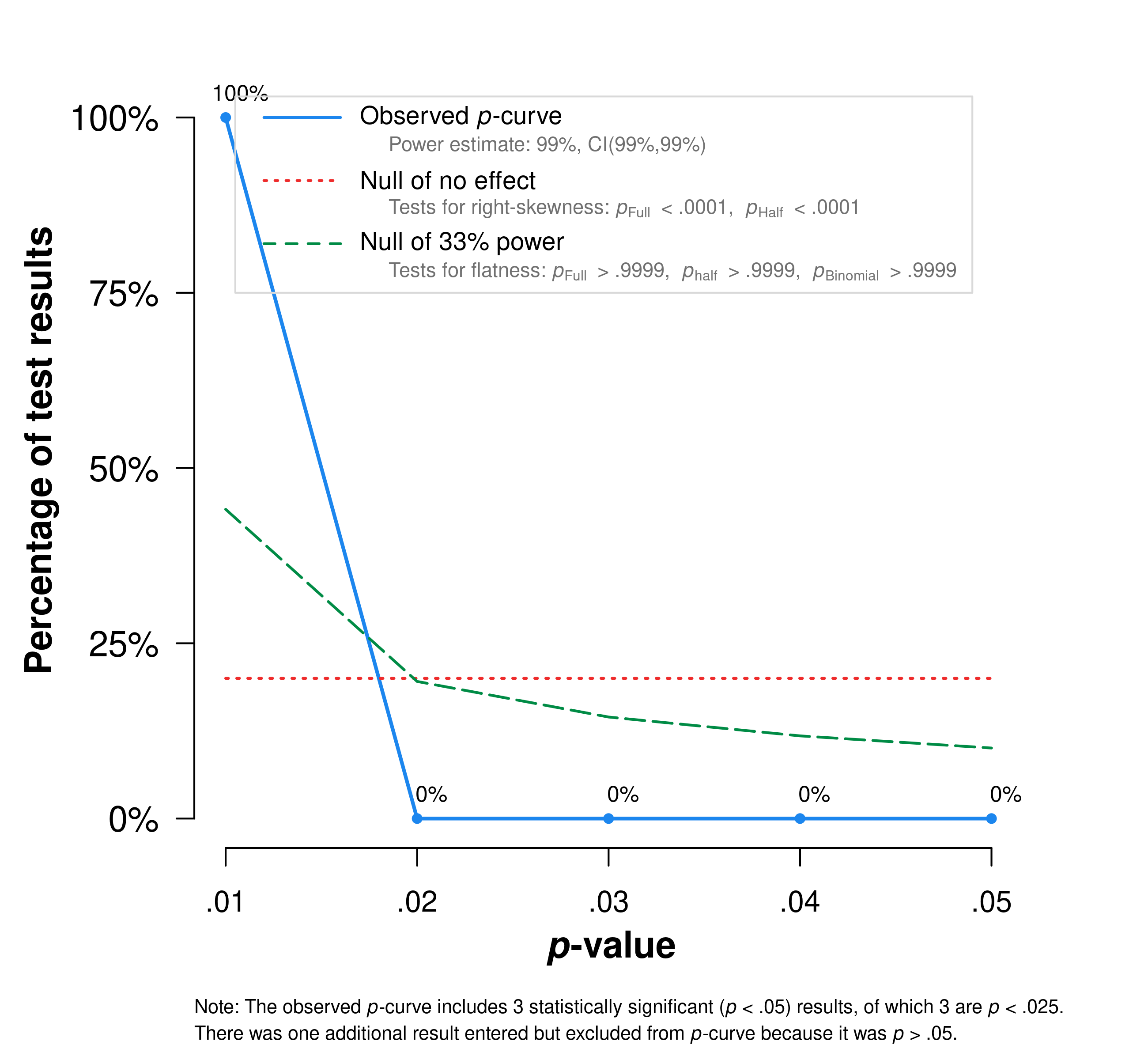
*

***Ingroup identification***

The forest plot of the ingroup identification effects shows that workplace identification was the only negative effect size, but that this study was also given slightly less weight than the other three (see Figure S6). The Bayesian posterior predictive distribution confirmed that the model converged successfully (Figure S7), and effect sizes were normally distributed around the obtained effect size (Figure S8). However, there was some notable positive skew in the between-study variance distribution (Figure S9). The sensitivity analysis confirmed that the estimated within-study effects were appropriate, but both the effect size heterogeneity, I^2^ = 81.41%, and between study variance, τ^2^ = .04, were found to be considerably high. The *P-Curve* analysis indicated that the significant effect sizes contained evidential value, *Z* = -1.87, *p* = .031, but showed that the average estimated power level was only 70%, 95% CIs [7%, 98%], with a notable cluster of effects around the *p* = .03 region of the *P-Curve* (see Figure S10). This suggested that the evidential value was not certain.^[[3]](#footnote-3)^

**Figure S6**

*Forest plot of the ingroup identification effect sizes*


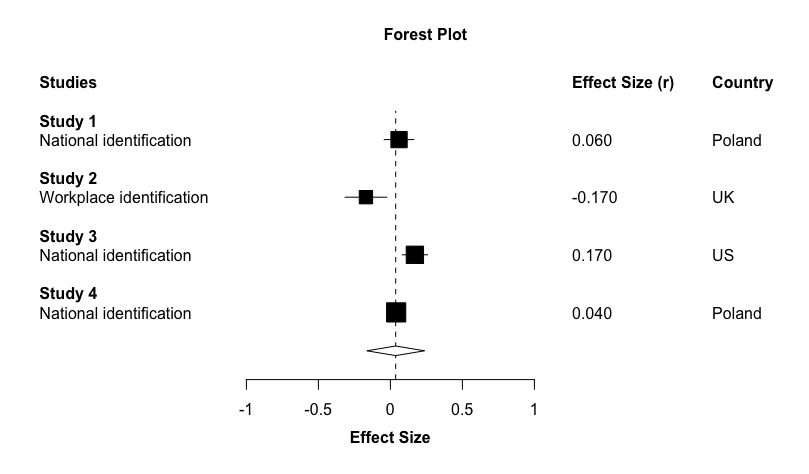


**Figure S7**

*Bayesian posterior predictive distribution for ingroup identification (iterations: 6,000)*


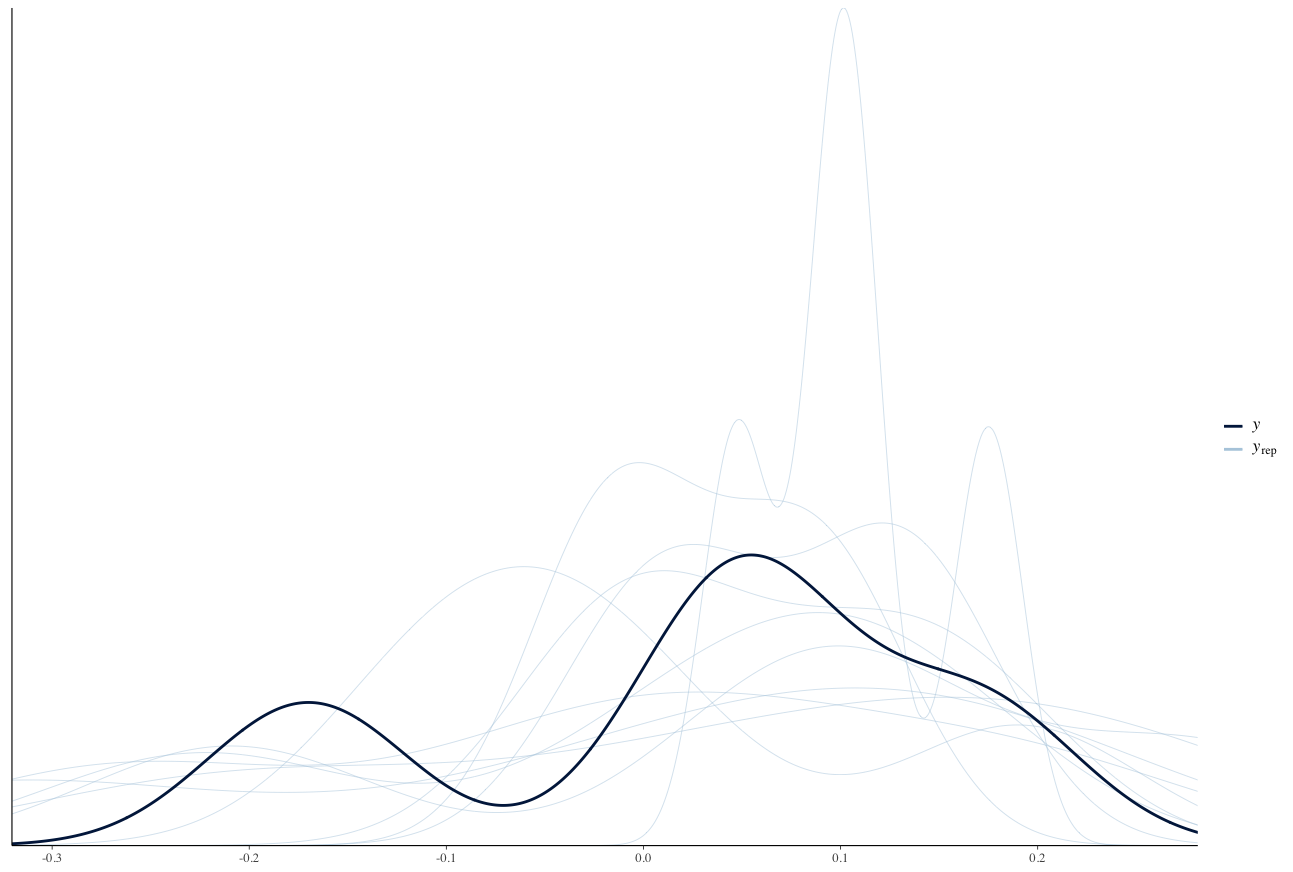


**Figure S8**

*Effect size distribution for ingroup identification* (central *r* = .02)


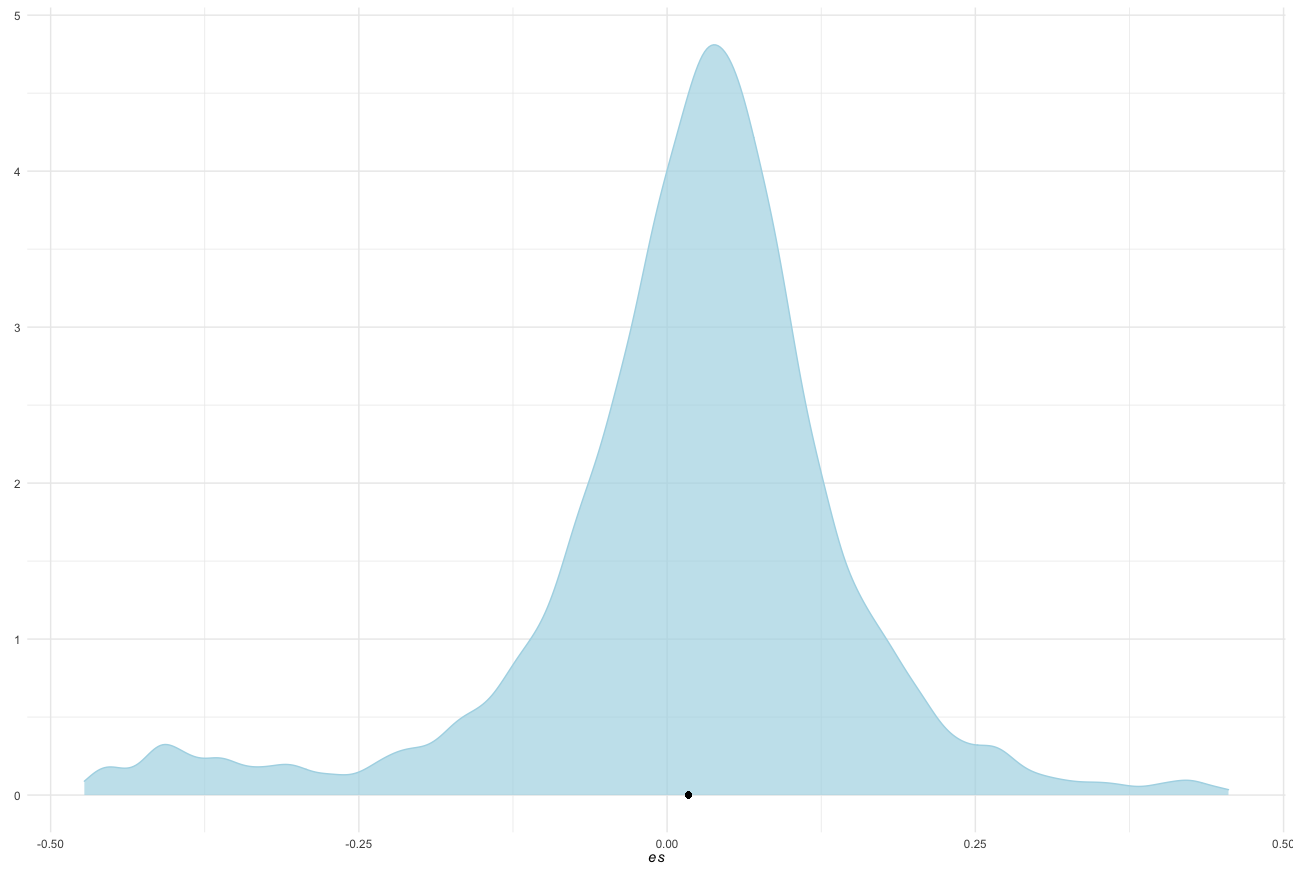


**Figure S9**

*Between-study variance distribution for ingroup identification* (central τ = .20)


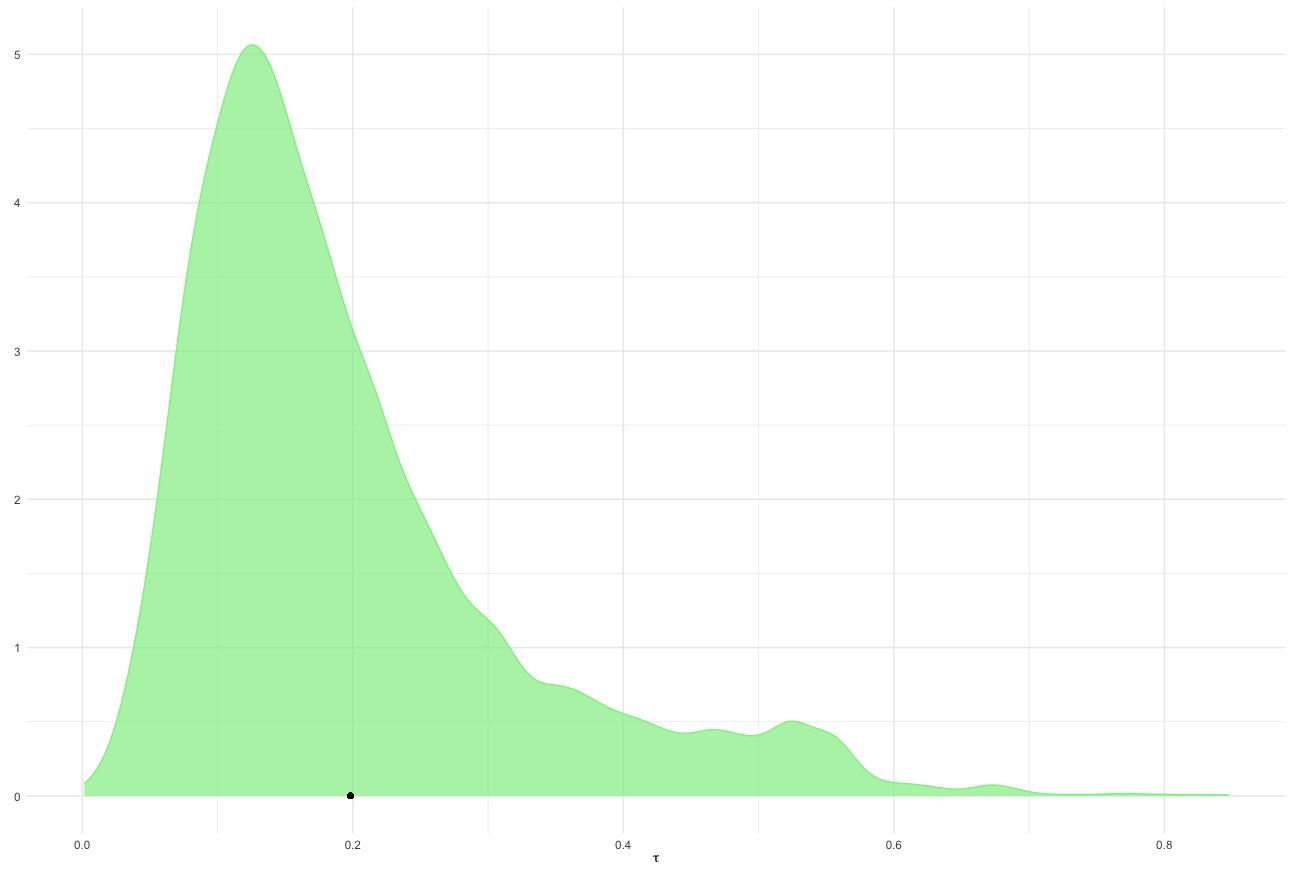


**Figure S10**

*P-Curve for ingroup identification*


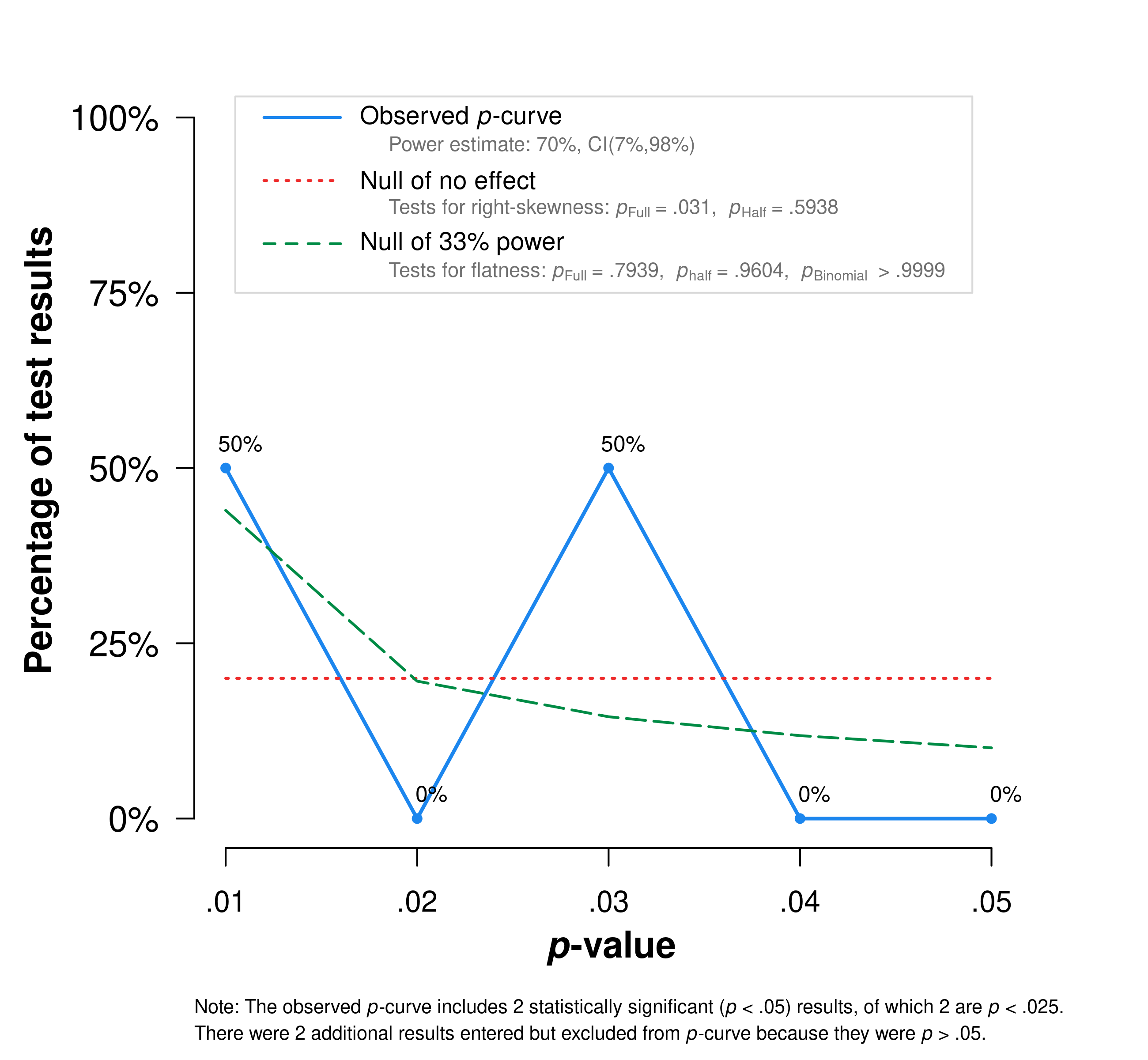


***Right-wing ideology***

The forest plot of the right-wing ideology effects showed that the effect sizes were generally uniform (Figure S11), and the Bayesian posterior predictive distribution confirmed that the model converged successfully (Figure S12). There was some notable kurtosis in the effect size distribution (Figure S13), alongside some positive skew in the between-study variance distribution (Figure S14). The sensitivity analysis confirmed that the estimated within-study effects were appropriate, and the effect size heterogeneity was suitable, I^2^ = 37.76%, with reasonable between study variance, τ^2^ = .01. The *P-Curve* analysis indicated that the significant effects contained evidential value, *Z* = -6.12, *p* < .001, and the average estimated power level was 99%, 95% CIs [94%, 99%] (see Figure S15).^[[4]](#footnote-4)^

**Figure S11**

*Forest plot of the right-wing ideology effect sizes*


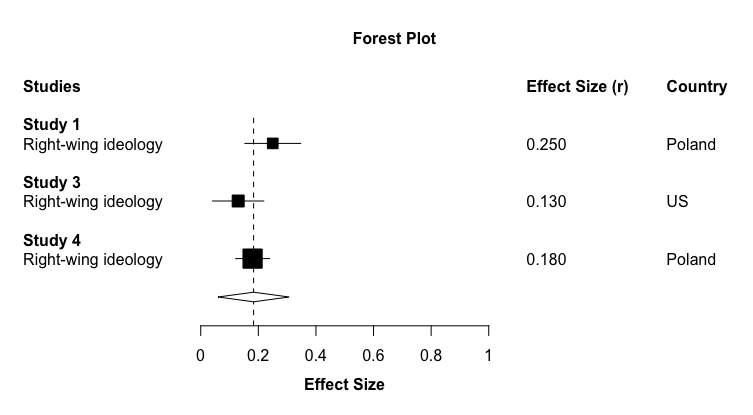


**Figure S12**

*Bayesian posterior predictive distribution for right-wing ideology (iterations: 40,000)*


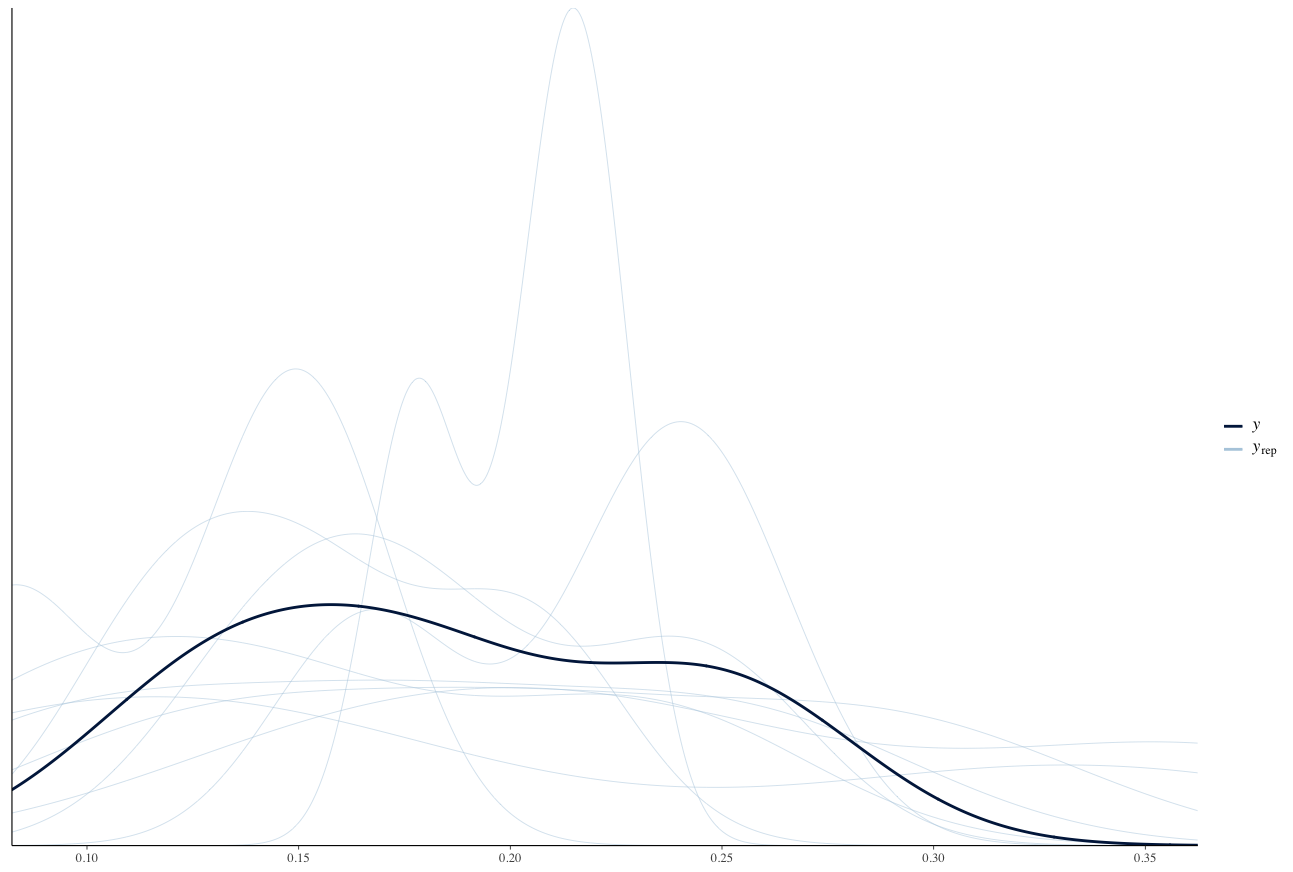


**Figure S13**

*Effect size distribution for right-wing ideology* (central *r* = .19)


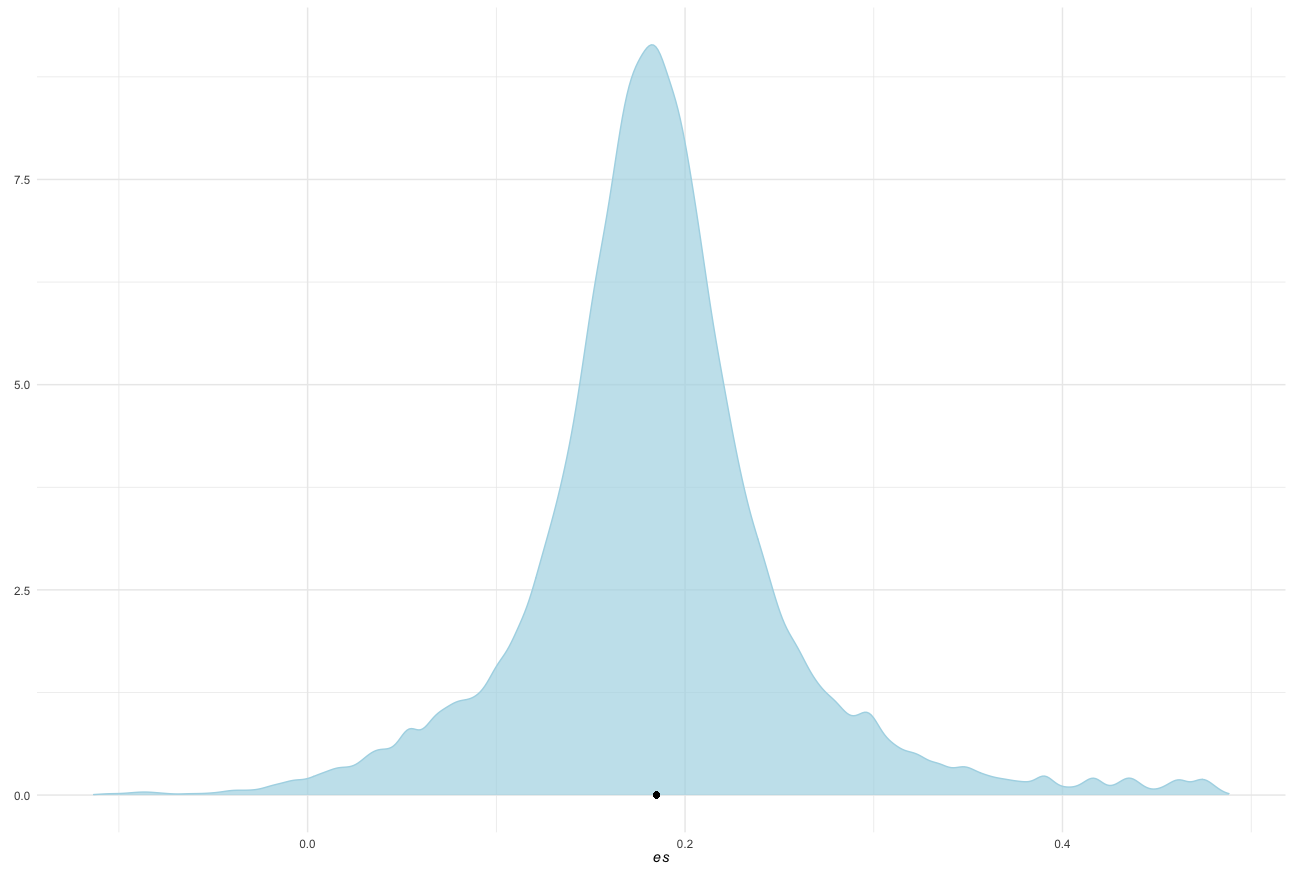


**Figure S14**

*Between-study variance distribution for right-wing ideology* (central τ = .10)


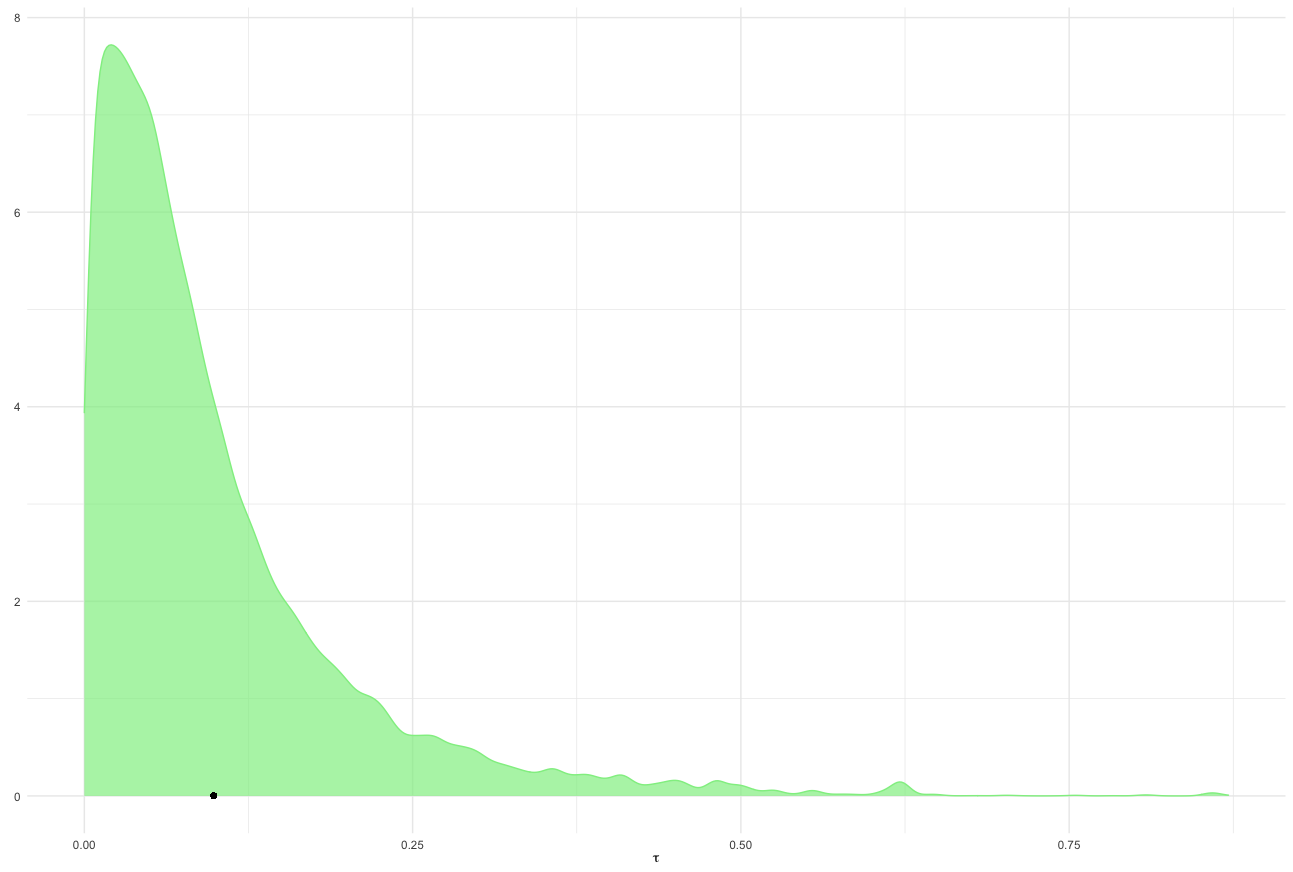


**Figure S15**

*P-Curve for right-wing ideology*


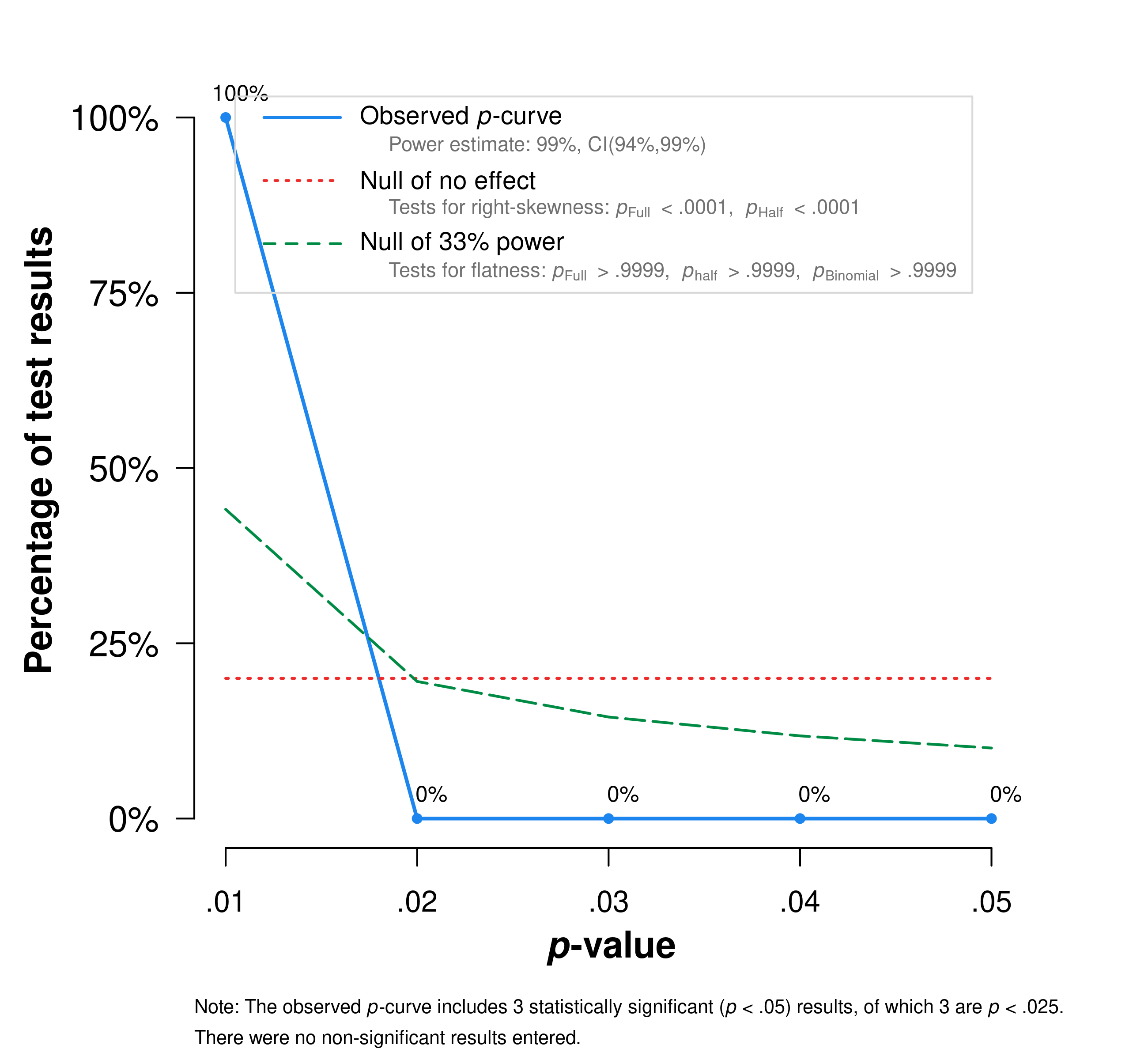


**Pilot Study Materials**

Note that the order of presentation of the collective narcissism and identification scales was counterbalanced.

CONTEMPORARY SOCIAL PROBLEMS

The study concerns the attitudes of Polish society in relation to current social problems. We are interested in Poles' attitudes towards themselves and opinions on issues that are currently the subject of public debate. We are interested in a wide range of problems, in this study we will ask you to express your opinion on one of them.

**Please read the following statements carefully, then mark on the scale how much you agree with them. Please provide your answer using a scale from 1 - definitely not to 5 - definitely yes.**

| 1 2 3 4 5  Definitely Definitely  not yes | |
| --- | --- |
| 1. 1. I wish other countries would more quickly recognize the authority of Poles. | 1 2 3 4 5 |
| 1. 2. Not many people seem to fully understand the importance of Poles. | 1 2 3 4 5 |
| 1. 3. It really makes me angry when others criticize Polish people. | 1 2 3 4 5 |
| 1. 4. If Poles had a major say in the world, the world would be a much better place. | 1 2 3 4 5 |
| 1. 5. I will never be satisfied until Poles get the recognition they deserve | 1 2 3 4 5 |
| 1. 6. I have a lot in common with other Polish people. | 1 2 3 4 5 |
| 1. 7. I feel strong ties to other Polish people. | 1 2 3 4 5 |
| 8. Being Polish is an important reflection of who I am. | 1 2 3 4 5 |
| 9. In general, I am glad to be Polish. | 1 2 3 4 5 |
| 10. I don’t feel good about being Polish. | 1 2 3 4 5 |

**Control condition**

**Personal Belief Survey**

The following questions measure personal beliefs. These questions relate to YOUR beliefs on each of the topics listed. There are no right or wrong answers here, so please try to be as honest as possible. Put an "X" next to the answer that best describes your beliefs. If you answered "YES" to any of these questions, please provide the reason why you believe the statements are true.

**1) I think that the colour blue looks great on most people.**  _____ YES _____ NO

IF YES, WHY?:

**2) I think that chocolate is the best flavour for ice cream.** _____ YES _____ NO

IF YES, WHY?:

**3) I think that winter is the most satisfying season during the year.**  _____ YES _____ NO

IF YES, WHY?:

**4) I think that the most aromatic trees in the world are pine trees.** _____ YES _____ NO

IF YES, WHY?:

**5) I think that cooking is an important skill to possess.** _____ YES _____ NO

IF YES, WHY?:

**6) I think that house plants help to brighten a home.** _____ YES _____ NO

IF YES, WHY?:

**7) I think that sewing is an important skill to possess.**  _____ YES _____ NO

IF YES, WHY?:

**8) I think that the beach is a great place to vacation.** _____ YES _____ NO

IF YES, WHY?:

**9) I think that the subway is the best form of public transportation.** _____ YES _____ NO

IF YES, WHY?:

**10) I think that fruit makes the best dessert.** _____ YES _____ NO

IF YES, WHY?:

**Experimental condition**

**Personal Properties Scale**

The following questions measure the level of friendliness towards others. These questions relate to the behaviours that YOU have performed towards others. As you read each question, please try to recall situations in which you did each of these behaviours for the other person. There are no right or wrong answers here, so please try to be as honest as possible. Put an "X" next to the answer that best describes your behaviour towards others. If you answered "YES" to any of these questions, please provide a brief example of the last time you acted like this.

**1) Have you ever forgiven another person when they have hurt you?**  _____ YES _____ NO

IF YES, EXAMPLE:

**2) Have you ever been considerate of another person's feelings?** _____ YES _____ NO

IF YES, EXAMPLE:

**3) Have you ever been concerned with the happiness of another person?** _____ YES _____ NO

IF YES, EXAMPLE:

**4) Have you ever looked out for another person's interests before your own?**  _____ YES _____ NO

IF YES, EXAMPLE:

**5) Have you ever been generous and selfless to another person?** _____ YES _____ NO

IF YES, EXAMPLE:

**6) Have you ever attended to the needs of another person?** _____ YES _____ NO

IF YES, EXAMPLE:

**7) Have you ever tried not to hurt the feelings of another person?** _____ YES _____ NO

IF YES, EXAMPLE:

**8) Have you ever felt satisfied when you've helped another person?**  _____ YES _____ NO

IF YES, EXAMPLE:

**9) Have you ever gone out of your way to help a friend even at the expense of your own happiness?**

_____ YES _____ NO

IF YES, EXAMPLE:

**10) Have you ever found ways to help another person who was less fortunate than yourself?**

_____ YES _____ NO

IF YES, EXAMPLE:

**Please read carefully the following statements regarding one of the issues under public debate.**

In August 2019, the Polish media received information from the journalists of *Gazeta Wyborcza* and *TVN24*, indicating that the Polish Central Anticorruption Bureau (CBA) was to purchase modern and very advanced software called "Pegasus". "Pegasus" is advertised as a tool to counter terrorism and fight crime. The purchase in the amount of PLN 25 million was made by the Crime Victims Assistance Fund. According to experts, the "Pegasus" system is one of the most modern programs used to control calls and messages sent (e.g. from a mobile phone) by specific people. They also indicate that there is a danger that "Pegasus" can access any data from the phone, and nothing limits it in terms of downloading and using it. There were even suggestions in the media that "Pegasus" could be used to surveillance representatives of specific social groups. Officially, the Polish government has neither confirmed nor denied the information about the purchase of "Pegasus" by the CBA.

**What is your opinion on Poland's actions in this matter? Please provide your answer using a scale from 1 - definitely not to 5 - definitely yes.**

| 1 2 3 4 5  Definitely Definitely  not yes | |
| --- | --- |
| 1. I think that Polish special forces should have the "Pegasus" software. | 1 2 3 4 5 |
| 2. Polish state needs systems such as "Pegasus" even at the cost of violating the privacy of citizens. | 1 2 3 4 5 |
| 3. If the information about the purchase of the "Pegasus" system by the CBA were confirmed by the Polish government, I would support such a decision of Polish services. | 1 2 3 4 5 |

Finally, please provide some information about yourself:

- Age: ....................
- Gender (Please circle the answer): W M
- Education (Please circle the answer):

1. primary
2. vocational
3. secondary
4. higher

- We ask you to identify your political views::

1. definitely leftist
2. leftist
3. leftist
4. centrist
5. moderately right-wing
6. right wing
7. definitely right-wing

- What party did you vote for during last parliamentary elections in 2019?:

1. Law and Justice Party
2. Citizens' Coalition
3. Polish People's Party
4. Democratic Left Alliance
5. Confederation of Freedom and Independence
6. Non-Party Coalition and Local Government
7. Other – ………………………..
8. I did not vote in parliamentary elections.

THANK YOU FOR PARTICIPATING IN THE STUDY!

**Study 1 Materials**

**Measures**

**Collective narcissism**. Study 1 used the same items of Golec de Zavala and colleagues (2009) Collective Narcissism Scale as the Pilot study:

1. I wish other countries would more quickly recognize the authority of Poles.
2. Not many people seem to fully understand the importance of Poles.
3. It really makes me angry when others criticize Polish people.
4. If Poles had a major say in the world, the world would be a much better place.
5. I will never be satisfied until Poles get the recognition they deserve..

**National identification** was measured with items adapted from Cameron (2004). Study 2 used the shorter version of the original 12-item scale (items 1, 2, 7, 9 & 11).

1. I have a lot in common with other Polish people.
2. I feel strong ties to other Polish people.
3. Being Polish is an important reflection of who I am.
4. In general, I am glad to be Polish.
5. I don’t feel good about being Polish.

**Support for in-group conspiracy** was measured with three items. Participants were asked to indicate the extent to which they agree with those items on a scale from 1= *definitely not* to 7= *definitely yes.*

“If you held a position in the government, would you support the rapid response of special services by:”

1. being able to wiretap Polish citizens?
2. Internet surveillance without obtaining the consent of Polish citizens?
3. the spread of false information if the situation required it?

**Political ideology** was measured with a single-item measure “We ask you to identify your political views”, from 1=*definitely leftist,* to 7=*definitely right-wing.*

**Education** options were “basic”, “vocational”, “secondary”, or “higher”.

**Study 2 Materials**

Study Information

We invite you to participate in a study on your workplace and your interactions with others. The project is led by (BLINDED) and (BLINDED) at (BLINDED).
   You will be asked to answer a few short questions about you feelings related to your workplace. The study should take around 2-4 minutes to complete.
You need to be 18 to take part. There are no risks or discomfort associated with taking part in the study.   Any responses you provide will be treated confidentially.  Any publication resulting from this work will report only aggregated findings or fully anonymised examples that will not identify you.
Any responses you provide will be completely anonymous—you will be asked to generate a participant code which will be kept with your responses in case of withdrawal of data. Only the research team will have access to the participant codes. The rest of your responses (anonymous) may be used by the research team, shared with other researchers, or made available in an online data repository.  
Remember that participation in this research study is completely voluntary. Even after you agree to participate and begin the study, you are still free to withdraw at any time and for any reason. Please note that once your data have been included in published analysis or data repositories, it cannot be withdrawn.
If you would like a copy of this information sheet to keep, please ask the researcher. If you have any complaints or concerns about this research, you can direct these, in writing, to (BLINDED).

**Consent**
 
 Please confirm that you agree with the information presented on the information sheet and with the statements listed below.

| 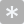 |
| --- |

I confirm that I have read and understand the information sheet for the above study.  I have had the opportunity to consider the information, ask questions and have had these answered satisfactorily.
I understand that my participation is voluntary and that I am free to withdraw at any time without giving any reason.
I understand that my data will be treated confidentially and any publication resulting from this work will report only data that does not identify me. My anonymised responses, however, may be shared with other researchers or made available in online data repositories.
I freely agree to participate in this study.

- Agree

End of Block: Information

Start of Block: Prolific ID

Please write your Prolific Id below:

________________________________________________________________

End of Block: Prolific ID

Start of Block: Collective Narcissism

Please think about **the team you work in**. Please indicate your agreement (1 = Strongly disagree, 7 = Strongly agree) with the following statements about your workplace team.

My team deserves special treatment.

- Strongly disagree
- Disagree
- Somewhat disagree
- Neither agree nor disagree
- Somewhat agree
- Agree
- Strongly agree

Not many people seem to understand the importance of my team.

- Strongly disagree
- Disagree
- Somewhat disagree
- Neither agree nor disagree
- Somewhat agree
- Agree
- Strongly agree

It really makes me angry when others criticise my team.

- Strongly disagree
- Disagree
- Somewhat disagree
- Neither agree nor disagree
- Somewhat agree
- Agree
- Strongly agree

If my team had a major say in our workplace, it would be a much better place.

- Strongly disagree
- Disagree
- Somewhat disagree
- Neither agree nor disagree
- Somewhat agree
- Agree
- Strongly agree

I will never be satisfied until my team gets the recognition it deserves.

- Strongly disagree
- Disagree
- Somewhat disagree
- Neither agree nor disagree
- Somewhat agree
- Agree
- Strongly agree

End of Block: Collective Narcissism

Start of Block: Cameron Team Identification

Please think about the team you work in. Please indicate your agreement (1 = Strongly disagree, 7 = Strongly agree) with the following statements about your workplace team.

I have a lot in common with other team members.

- Strongly disagree
- Disagree
- Somewhat disagree
- Neither agree nor disagree
- Somewhat agree
- Agree
- Strongly agree

I feel strong ties to other team members.

- Strongly disagree
- Disagree
- Somewhat disagree
- Neither agree nor disagree
- Somewhat agree
- Agree
- Strongly agree

I find it difficult to form a bond with other team members.

- Strongly disagree
- Disagree
- Somewhat disagree
- Neither agree nor disagree
- Somewhat agree
- Agree
- Strongly agree

I don't feel a sense of being 'connected' with other team members.

- Strongly disagree
- Disagree
- Somewhat disagree
- Neither agree nor disagree
- Somewhat agree
- Agree
- Strongly agree

I often think about the fact that I am a member of my team.

- Strongly disagree
- Disagree
- Somewhat disagree
- Neither agree nor disagree
- Somewhat agree
- Agree
- Strongly agree

The fact that I am a member of my team rarely enters my mind.

- Strongly disagree
- Disagree
- Somewhat disagree
- Neither agree nor disagree
- Somewhat agree
- Agree
- Strongly agree

Being a member of my team is an important reflection of who I am.

- Strongly disagree
- Disagree
- Somewhat disagree
- Neither agree nor disagree
- Somewhat agree
- Agree
- Strongly agree

In general, I am glad to be a member of my team.

- Strongly disagree
- Disagree
- Somewhat disagree
- Neither agree nor disagree
- Somewhat agree
- Agree
- Strongly agree

I often regret that I am a member of my team.

- Strongly disagree
- Disagree
- Somewhat disagree
- Neither agree nor disagree
- Somewhat agree
- Agree
- Strongly agree

I don’t feel good about being a member of my team.

- Strongly disagree
- Disagree
- Somewhat disagree
- Neither agree nor disagree
- Somewhat agree
- Agree
- Strongly agree

Generally, I feel good when I think about myself as a member of my team.

- Strongly disagree
- Disagree
- Somewhat disagree
- Neither agree nor disagree
- Somewhat agree
- Agree
- Strongly agree

Overall, being a member of my team has very little to do with how I feel about myself.

- Strongly disagree
- Disagree
- Somewhat disagree
- Neither agree nor disagree
- Somewhat agree
- Agree
- Strongly agree

End of Block: Cameron Team Identification

Start of Block: Interaction?

Do you ever interact with **members of other teams similar to yours** (either within your organisation or in another organisation)?

- Yes -> Participants directed to take part in a different study
- No -> Participants took part in our study

End of Block: Interaction?

Start of Block: Ingroup Conspiracy Intentions

Imagine you've learned that some of your friends secretly coordinated to engage in activities that would help you gain advantage over **other members of your team**. To what extent would you be willing to join the following activities in your workplace:

Control other team members' computers without their knowledge.

- Definitely not
- Probably not
- Might or might not
- Probably yes
- Definitely yes

Monitor other team members' web activity without their knowledge.

- Definitely not
- Probably not
- Might or might not
- Probably yes
- Definitely yes

Secretly spread false information about team members to gain influence.

- Definitely not
- Probably not
- Might or might not
- Probably yes
- Definitely yes

Secretly record audio of other team members without their knowledge.

- Definitely not
- Probably not
- Might or might not
- Probably yes
- Definitely yes

Engage in coordinated efforts to promote loyal colleagues, even if they are not performing well.

- Definitely not
- Probably not
- Might or might not
- Probably yes
- Definitely yes

End of Block: Ingroup Conspiracy Intentions

Start of Block: Demographics

**Demographics**
 
Please provide us with basic information about you:

Age

|  | 18 | 26 | 34 | 43 | 51 | 59 | 67 | 75 | 84 | 92 | 100 |
| --- | --- | --- | --- | --- | --- | --- | --- | --- | --- | --- | --- |

| Years | 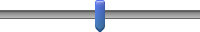 |
| --- | --- |

Gender

- Male
- Female
- Transgender
- Non-Binary
- Other
- Prefer not to say

Highest level of completed education

- GCSE
- A level/IB
- Bachelor's
- Master's
- Post-Graduate/Higher

End of Block: Demographics

Start of Block: Debrief

**Debriefing**

 You participated in a study that examines factors that influence people’s intentions towards their own and others' workplace teams. As a participant, you were asked questions that measured the extent to which you identify with your workplace team. You were then asked whether you frequently interact with other workplace teams. If you don't, you were asked about your intentions to engage in secretive behaviours in your workplace. If you do, you were presented with questions measuring the way you treat members of your own, or another workplace team. Our main prediction is that excessive identification with your team might paradoxically lead to treating others instrumentally.
   There are no right or wrong answers and you can be sure of a confidential treatment of your responses. If you have any further questions, or you wish to withdraw from the study please use the contact details given below. Once again, thank you for your participation. (BLINDED)

End of Block: Debrief

**Study 3 Materials**

Note that identical surveys were conducted separately among Clinton vs Trump supporters (pre-screened through *Prolific*).

Start of Block: Intro

**Study Information**
  We invite you to participate in a study on beliefs about the US. The project is led by (BLINDED) and (BLINDED) at (BLINDED). You will be asked to answer a few short questions about your feelings and attitudes related to the US in general and the US government. The study should take around 2-4 minutes to complete.   You need to be 18 to take part. There are no risks or discomfort associated with taking part in the study.   Any responses you provide will be treated confidentially.  Any publication resulting from this work will report only aggregated findings or fully anonymised examples that will not identify you.   Any responses you provide will be completely anonymous—you will be asked to provide your Prolific ID which will be kept with your responses in case of withdrawal of data. Only the research team will have access to the codes. The rest of your responses (anonymous) may be used by the research team, shared with other researchers, or made available in an online data repository.    Remember that participation in this research study is completely voluntary. Even after you agree to participate and begin the study, you are still free to withdraw at any time and for any reason. Please note that once your data have been included in published analysis or data repositories, it cannot be withdrawn.   If you would like a copy of this information sheet to keep, please ask the researcher. If you have any complaints or concerns about this research, you can direct these, in writing, to (BLINDED)

**Consent**
  
 Please confirm that you agree with the information presented on the information sheet and with the statements listed below.

| 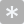 |
| --- |

I confirm that I have read and understand the information sheet for the above study.  I have had the opportunity to consider the information, ask questions and have had these answered satisfactorily.   I understand that my participation is voluntary and that I am free to withdraw at any time without giving any reason.   I understand that my data will be treated confidentially and any publication resulting from this work will report only data that does not identify me. My anonymised responses, however, may be shared with other researchers or made available in online data repositories.   I freely agree to participate in this study.

- Agree

End of Block: Intro

Start of Block: Prolific ID

| 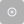 |
| --- |

**Prolific ID**   Before you start, please provide your unique *Prolific* ID. This will allow you to withdraw your answers later on if so desired.

________________________________________________________________

End of Block: Prolific ID

Start of Block: Short CN

Please indicate your agreement with the following statements about the US, from 1 (Strongly disagree) to 5 (Strongly agree).

The US deserves special treatment.

- Strongly disagree
- Disagree
- Neither agree nor disagree
- Agree
- Strongly agree

Not many people seem to fully understand the importance of the US.

- Strongly disagree
- Disagree
- Neither agree nor disagree
- Agree
- Strongly agree

It really makes me angry when others criticize the US.

- Strongly disagree
- Disagree
- Neither agree nor disagree
- Agree
- Strongly agree

If the US had a major say in the world, the world would be a much better place.

- Strongly disagree
- Disagree
- Neither agree nor disagree
- Agree
- Strongly agree

I will never be satisfied until the US gets the recognition it deserves.

- Strongly disagree
- Disagree
- Neither agree nor disagree
- Agree
- Strongly agree

End of Block: Short CN

Start of Block: Identification

Now please indicate your agreement with the next following statements about the US, from 1 (Strongly disagree) to 7 (Strongly agree).

I am glad to be American.

- Strongly Disagree
- Disagree
- Neither agree nor disagree
- Agree
- Strongly agree

I think that Americans have a lot to be proud of.

- Strongly Disagree
- Disagree
- Neither agree nor disagree
- Agree
- Strongly agree

It is pleasant to be American.

- Strongly Disagree
- Disagree
- Neither agree nor disagree
- Agree
- Strongly agree

Being American gives me a good feeling.

- Strongly Disagree
- Disagree
- Neither agree nor disagree
- Agree
- Strongly agree

End of Block: Identification

Participants were then presented with a combination of four belief and four intentions items (shown below). As each of the two belief blocks had an adapted counterpart intentions block, participants were only presented with the intentions items that were not based on the belief items they were going to see. That is, if participants were presented with Block 1 of the belief items, they would only see the intentions items from Block 1 (and the same for Block 2 of beliefs and intentions; see below). The combination of blocks presented was randomised, and the order of beliefs and intentions blocks were randomly presented.

For the belief items, participants were presented with the following text: “Now, please indicate your agreement with the following beliefs **about the US government**, from definitely *not true* (1) to *definitely true* (5).” Then, participants were randomly presented one of the two 4-item belief blocks:

**Block 1**

1. A small, secret group of people is responsible for making all major world decisions, such as going to war.
2. The government permits or perpetrates acts of terrorism on its own soil, disguising its involvement.
3. New and advanced technology which would harm current industry is being suppressed.
4. The government uses people as patsies to hide its involvement in criminal activity.

**Block 2**

1. Experiments involving new drugs or technologies are routinely carried out on the public without their knowledge or consent.
2. The government is involved in the murder of innocent citizens and/or well-known public figures, and keeps this a secret.
3. The spread of certain viruses and/or diseases is the result of the deliberate, concealed efforts of some organization.
4. A lot of important information is deliberately concealed from the public out of self-interest.

For the intentions items, participants were presented with the following text: “Now, please indicate the likelihood that you would engage in these behaviours **if you worked for or with the US government**, from *I would never do this* (1) to *I would definitely do this* (5).” Then, participants were randomly presented with one of the two 4-item blocks:

**Block 1**

1. If it was necessary, I would carry out experiments involving new drugs or technologies on the public without their knowledge or consent.
2. If asked, I would aid organizations in concealing efforts that could lead to the spread of certain viruses and/or diseases.
3. If I was working for the government, I would help deliberately conceal some information from the public.
4. If necessary, I would support government-sponsored secret murder of innocent citizens and/or well-known public figures.

**Block 2**

1. If I was a member of the government, I would form a small, secret group of people to make all major world decisions, such as going to war.
2. If it was necessary, I would work with the government to carry out acts of terrorism on my own soil, disguising our involvement.
3. If I was in charge, I would suppress new and advanced technology that could harm current industry.
4. I would aid the government in using people as patsies to hide its involvement in criminal activity.

End of Block: Intentions-belief 2

Start of Block: Dems

**Demographics**
 
Please provide us with basic information about you:

Age

|  | 18 | 26 | 34 | 43 | 51 | 59 | 67 | 75 | 84 | 92 | 100 |
| --- | --- | --- | --- | --- | --- | --- | --- | --- | --- | --- | --- |

| Years | 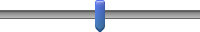 |
| --- | --- |

Gender

- Male
- Female
- Transgender
- Non-Binary
- Other
- Prefer not to say

Highest level of completed education^[[5]](#footnote-5)^

- GCSE
- A level/IB
- Bachelor's
- Master's
- Post-Graduate/Higher

Overall, where would you place yourself, on the following scale of liberalism-conservatism?

- Extremely liberal 1
- Liberal 2
- Centrist 3
- Conservative 4
- Extremely conservative 5

End of Block: Dems

Start of Block: Debrief

**Debriefing**
  You participated in a study that examines factors that influence people’s conspiratorial beliefs about, and intentions to conspire with the US government. As a participant, you were asked questions that measured your identification with the US. You were then asked the extent to which you would endorse and intend to take part in conspiratorial actions of the US government. Our main prediction is that insecure US national identification will positively predict US government conspiracy beliefs indirectly through personal willingness to conspire.   There are no right or wrong answers and you can be sure of a confidential treatment of your responses. If you have any further questions, or you wish to withdraw from the study please use the contact details given below. Once again, thank you for your participation.
(BLINDED)

End of Block: Debrief

**Study 4 Materials**

**Measures**

**Collective narcissism.** As in the Pilot study and in Study 1, we used the following five items:

1. I wish other countries would more quickly recognize the authority of Poles.
2. Not many people seem to fully understand the importance of Poles.
3. It really makes me angry when others criticize Polish people.
4. If Poles had a major say in the world, the world would be a much better place.
5. I will never be satisfied until Poles get the recognition they deserve.

**National identification** was measured with items adapted from (Cameron, 2004). Study 4 used the shorter version of the original 12-item scale:

1. I have a lot in common with other Polish people.
2. I feel strong ties to other Polish people.
3. Being Polish is an important reflection of who I am.
4. In general, I am glad to be Polish.
5. I don’t feel good about being Polish.

**Social Dominance Orientation** was measured with Pratto and colleagues’ (2013) Polish version of the four-item Short Social Dominance Orientation (SSDO).

1. In setting priorities, we must consider all groups.
2. We should not push for group equality.
3. Group equality should be our ideal.
4. Superior groups should dominate inferior groups.

**Dark Triad Personality Traits** were measured using Czarna and colleagues’ (2016) Polish translation of the 12-item Dirty Dozen (DTDD-P) scale.

*Individual Narcissism*

1. I tend to seek prestige or status.
2. I tend to expect special favors from others.
3. I tend to want others to admire me.
4. I tend to want others to pay attention to me.

*Psychopathy*

1. I tend to lack remorse.
2. I tend to be callous or insensitive.
3. I tend to not be too concerned with morality or the morality of my actions.
4. I tend to be cynical.

*Machiavellianism*

1. I tend to manipulate others to get my way.
2. I tend to exploit others towards my own end.
3. I have used deceit or lied to get my way.
4. I have used flattery to get my way.

**Conspiracy intentions** were measured with three items. Participants were asked to indicate the extent to which they agree with those items on a scale from 1= *definitely not* to 7= *definitely yes.*

“If you held a position in the government, would you support the rapid response of special services by:”

1. being able secretly to wiretap Polish citizens?
2. Internet surveillance without obtaining the consent of Polish citizens?
3. the spread of false information if the situation required it?

**Perceived ingroup typicality** was measured with three items. Participants stated their answers on a scale from 1= *not at all similar to a typical Pole* to 7= *very similar to a typical Pole.*

"Sometimes the secret service is suspected of being allowed fast responses by the following actions. How similar to a typical Pole are, in your opinion, people who”:

1. need to be secretly wiretapped by secret services?
2. need to be surveilled without obtaining their consent?
3. about whom false information is spread if the situation requires it?

**Political ideology** was measured with a single-item measure “We ask you to identify your political views”, from 1=*definitely leftist,* to 7=*definitely right-wing.*

**Voting Intentions** were measured by asking participants “If parliamentary elections were held in Poland today, what party / election committee would you be willing to vote for?”. The options were “Law and Justice”, “Citizens’ Coalition”, “Poland 2050 Szymon Hołownia”, “Confederation of Freedom and Independence”, “Democratic Left Alliance”, “Polish People’s Party”, “Kukiz 15”, “Other”, “I would not vote”, and “I do not know”.

**Education** options were “primary”, “vocational”, “secondary”, or “higher”.

**References**

Bürkner, P-C. (2017). brms: An R package for Bayesian multilevel models using Stan. [Computer software manual]. <https://cran.r-project.org/package=brms> (R package version 1.9.0)

Cameron, J. E. (2004). A three-factor model of social identity. *Self and Identity*, *3*(3), 239–262. <https://doi.org/10.1080/13576500444000047>

Cohrs, J. C., Kielmann, S., Maes, J., & Moschner, B. (2005). Effects of right-wing authoritarianism and threat from terrorism on restriction of civil liberties. *Analyses of Social Issues and Public Policy*, *5*(1), 263-276. <https://doi.org/10.1111/j.1530-2415.2005.00071.x>

Golec de Zavala, A., Cichocka, A., Eidelson, R., & Jayawickreme, N. (2009). Collective narcissism and its social consequences. *Journal of Personality and Social Psychology*, *97*(6), 1074–1096. <https://doi.org/10.1037/a0016904>

Reed, M. B., & Aspinwall, L. G. (1998). Self-affirmation reduces biased processing of health-risk information. Motivation and Emotion, 22(2), 99–132. [https://doi.org/10.1023/A:1021463221281](https://psycnet.apa.org/doi/10.1023/A:1021463221281)

Reuters (2022). *Ruling party figures say Poland has Pegasus spyware.* <https://www.reuters.com/world/europe/ruling-party-figures-say-poland-has-pegasus-spyware-2022-01-07/>

Simonsohn, U., Nelson, L. D., & Simmons, J. P. (2014). P-curve: A key to the file-drawer. *Journal of Experimental Psychology: General*, *143*(2), 534–547. <https://doi.org/10.1037/a0033242>

1. For purposes of a different project, the study also used a between-groups experimental design, manipulating self-affirmation (kindness vs. control questionnaire; Reed and Aspinwall, 1998). Controlling for the effects of the manipulation did not affect the pattern of results (see Table S4). [↑](#footnote-ref-1)
2. Including the pilot study did not notably alter the results for collective narcissism, *r*=.25, 95% CI [.19, .31], *t*(3.43)=12.90, *p*<.001, but the Bayes Factor now provided strong evidence for its directional hypothesis, BF_10_=17.75. [↑](#footnote-ref-2)
3. Including the pilot study did not notably change the results for identification, *r*=.07, 95% CI [-.09, .22], *t*(3.94)=1.22, *p*=.291, BF_10_=0.10. [↑](#footnote-ref-3)
4. The effect size for political ideology became significant once the Pilot study was added to the analysis, *r*=.20, 95% CI [.11, .30], *t*(2.89)=7.11, *p*=.006, and the Bayes Factor now indicated weak evidence for its directional hypothesis, BF_10_=3.38. [↑](#footnote-ref-4)
5. This measure was removed from the main analyses because it was not applicable to the US context due to a mistake. Controlling for this measure did not significantly change the results. [↑](#footnote-ref-5)
